# Composite midazolam and 1′-OH midazolam population pharmacokinetic model for constitutive, inhibited and induced CYP3A activity

**DOI:** 10.1007/s10928-020-09704-1

**Published:** 2020-08-08

**Authors:** Sabrina T. Wiebe, Andreas D. Meid, Gerd Mikus

**Affiliations:** 1 Department of Clinical Pharmacology and Pharmacoepidemiology, University of Heidelberg, Im Neuenheimer Feld 410, 69120 Heidelberg, Germany; 2 Boehringer Ingelheim Pharma GmbH & Co. KG, Birkendorfer Str. 65, 88397 Biberach an der Riss, Germany

**Keywords:** CYP3A, Drug interactions, Midazolam, Population pharmacokinetics

## Abstract

CYP3A plays an important role in drug metabolism and, thus, can be a considerable liability for drug-drug interactions. Population pharmacokinetics may be an efficient tool for detecting such drug-drug interactions. Multiple models have been developed for midazolam, the typical probe substrate for CYP3A activity, but no population pharmacokinetic models have been developed for use with inhibition or induction. The objective of the current analysis was to develop a composite parent-metabolite model for midazolam which could adequately describe CYP3A drug-drug interactions. As an exploratory objective, parameters were assessed for potential cut-points which may allow for determination of drug-drug interactions when a baseline profile is not available. The final interaction model adequately described midazolam and 1′-OH midazolam concentrations for constitutive, inhibited, and induced CYP3A activity. The model showed good internal and external validity, both with full profiles and limited sampling (2, 2.5, 3, and 4 h), and the model predicted parameters were congruent with values found in clinical studies. Assessment of potential cut-points for model predicted parameters to assess drug-drug interaction liability with a single profile suggested that midazolam clearance may reasonably be used to detect inhibition (4.82–16.4 L/h), induction (41.8–88.9 L/h), and no modulation (16.4–41.8 L/h), with sensitivities for potent inhibition and induction of 87.9% and 83.3%, respectively, and a specificity of 98.2% for no modulation. Thus, the current model and cut-points could provide efficient and accurate tools for drug-drug liability detection, both during drug development and in the clinic, following prospective validation in healthy volunteers and patient populations.

**Electronic supplementary material:**

The online version of this article (10.1007/s10928-020-09704-1) contains supplementary material, which is available to authorized users.

## Introduction

Drug-drug interactions (DDIs) are a serious concern for drug developers, physicians, and patients, particularly with the increasing rate of polypharmacy [1–3]. Compounds may unintentionally interact with each other in numerous ways, as they may inhibit, inactivate, and/or induce drug metabolising enzymes of which another substance is a substrate [4, 5]. The family of haem-containing isoenzymes called cytochrome P450s (CYPs) metabolize the majority of xenobiotics [6]. In humans, one of the main CYPs, CYP3A, constitutes approximately 40% of the hepatic CYPs and almost 80% of intestinal CYPs [7, 8]. Accordingly, CYP3A is a main metabolizing enzyme of almost half of the currently marketed small molecule drugs [6, 9].

Given the potential DDI liability of newly developed drugs, the regulatory authorities (e.g. FDA, EMA and PMDA) have established thresholds based on in vitro data for when a clinical DDI trial should be conducted [10–12]. The thresholds are relatively conservative, in order to avoid false negative results and to protect patients. These conservative thresholds result in numerous DDI trials without significant effects [13, 14], which consume time and resources during drug development. This appears to be particularly likely when multiple mechanisms are present (e.g. induction plus inactivation) [15]. A tool that accurately predicts the overall CYP3A interaction liability of a drug early in development while limiting resources and time used, as well as reducing burden to subjects would be a great asset. Population pharmacokinetic modelling (PopPK) has been increasingly used for estimating various exposure parameters, including those resulting from DDIs. Amongst its advantages are the ability to be used with both extensive and sparse sampling, as well as a relatively simple implementation in early clinical development.

Midazolam is the main probe substrate used in CYP3A phenotyping studies [16] and is recommended by the regulatory authorities for CYP3A DDI studies [10–12, 17]. It is particularly well-suited for this purpose, given its almost complete metabolism via CYP3A. Interaction studies with ketoconazole, voriconazole and ritonavir, potent inhibitors of CYP3A, have found ~ 10–14-fold higher midazolam exposure [18, 19], while studies using carbamazepine, phenytoin or rifampicin, potent CYP3A inducers, reduce midazolam exposure to less than 10% of its baseline exposure [20, 21]. Such increases or decreases in exposure of CYP3A substrates can result in serious safety concerns or abolished efficacy, respectively. Midazolam’s primary metabolite, 1′-OH midazolam [22, 23], is produced via CYP3A4/5 metabolism and its glucuronide represents approximately 60–80% of the product recovered in urine following midazolam dosing [24, 25]. Thus, 1′-OH midazolam represents the metabolite most affected by CYP3A DDIs and its formation and/or clearance may provide additional information regarding the presence of a DDI compared to midazolam concentrations alone. This is supported by work examining a semi-physiological pharmacokinetic model where the time course of CYP3A inhibition processes could only be adequately captured with the incorportation of 1′-OH midazolam into the model [26].

Given the numerous DDI trials conducted during drug development based on in vitro findings, the development of an adequate composite midazolam/1′-OH midazolam PopPK model for describing various CYP3A activity conditions would facilitate DDI detection earlier in development while reducing burden to subjects and/or patients, as well as reducing time and resources required for study conduct. Specifically, a properly developed model can be used with limited sampling schemes during DDI assessment of potential CYP3A perpetrators, thereby reducing subject/patient time spent in the clinics, as well as reducing blood samples taken. An additional advantage may be the use with single midazolam/1′-OH midazolam profiles, if cut-points applied to a specific estimated parameter could be used to assess the presence of a DDI. Thus, the aims of the current analysis are: (1) to assess the ability of a PopPK model which combines midazolam and 1′-OH midazolam to describe pharmacokinetics both for constitutive CYP3A activity, as well as in the presence of a CYP3A-related DDI; (2) to examine the applicability of the model to limited sampling profiles; and (3) to identify possible parameter cut-points in healthy volunteers which may be used for determination of DDI presence based on a single profile.

## Methods

### Pharmacokinetic data

Midazolam and 1′-OH midazolam data were available from 10 previously conducted trials in healthy volunteers; data from seven of the studies was used for the model development set. These studies had various purposes, including examining CYP3A activity following different doses of midazolam alone, as well as in the presence of known inhibitors or inducers of CYP3A. A description of the available conditions, doses, and sampling times for each of the studies in both the development and validation sets is displayed in Table 1 and a brief description of the methodology used in each of the studies is provided in Online Resource 1, Table S1. In three of the studies, additional treatment arms were present which were not used for model development, but which were used as part of the external validation set, as they consisted of (i) midazolam administered in the presence of a substance (honey) which had been thought to potentially induce CYP3A activity, (ii) midazolam administered following withdrawal of a concomitantly administered inducer (St. John’s wort) and irreversible inhibitor (ritonavir), and (iii) midazolam administered following a single dose of an inducer/activator (efavirenz). To assess applicability to limited sampling schemes, three additional studies with limited sampling (pre-dose, 2, 2.5, 3, and 4 h) were included in the validation set; all treatment arms were assessed based on reported findings regarding CYP3A modulation [18, 27–29].

**Table 1:** Model dataset characteristics

Study	MDZ Alone	MDZ + Modulator	MDZ Doses	Route(s)	Sampling times [h]	References
K119^a,b^	X	X (RTV^a^/SJW^b^)	4 mg + 2 mg	po + iv	Pre-dose, 0.17, 0.33, 0.5, 0.75, 1, 1.5, 2, 3, 4, 5, 6, 6.25, 6.5, 6.67, 6.83, 7, 7.25, 7.5, 8, 9, 10, 12, 14, 24, 25	[28]
K155^a^	X	X (EFV)	3 mg	po	Pre-dose, 0.25, 0.5, 0.75, 1, 1.5, 2, 2.5, 3, 4, 6, 8, 10	[47]
K169^a,b^	X		4 mg + 2 mg	po + iv	Pre-dose, 0.17, 0.33, 0.5, 0.75, 1, 1.5, 2, 3, 4, 5, 6, 6.25, 6.5, 6.67, 6.83, 7, 7.25, 7.5, 8, 9, 10, 12, 14	[27]
K257^a,b^	X	X (VCZ/RTV)	3 mg	po	Pre-dose, 0.25, 0.5, 0.75, 1, 1.5, 2, 2.5, 3, 4, 5, 6, 8, 10^a^ or pre-dose, 2, 2.5, 3, 4^b^	[18]
K380^a^	X	X (VCZ)	1 mg (iv); 3 μg, 3 mg (po)	iv&po	Pre-dose, 0.083, 0.25, 0.5, 0.75, 1, 1.5, 2, 2.5, 3, 4, 6, 8, 10, 24	[48, 49]
K194^a,b^	X	X (EFV^b^)	4 mg + 2 mg	po + iv	Pre-dose, 0.17, 0.33, 0.5, 0.75, 1, 1.5, 2, 3, 4, 5, 6, 6.25, 6.5, 6.67, 6.83, 7, 7.25, 7.5, 8, 9, 10, 12, 14	[29]
K345^a^	X	X (keto)	100 μg, 1 mg, 3 μg, 30 μg, 3 mg	po	Pre-dose, 0.25, 0.5, 0.75, 1, 1.5, 2, 2.5, 3, 4, 6, 8, 10, 24	[50]
K292^b^	X	X (keto)	3 mg	po	Pre-dose, 2, 2.5, 3, 4	[37]
K342^b^	X	X (RTV)	3 mg	po	Pre-dose, 2, 2.5, 3, 4	[35]
K363^b^	X	X (RTV)	100 μg	po	Pre-dose, 2, 2.5, 3, 4	[36]

*EFV* efavirenz, *keto* ketoconazole, *MDZ* midazolam, *RTV* ritonavir, *VCZ* voriconazole, *SJW* St. John’s wort, *iv* intravenous dosing, *po per os* (oral) dosing

^a^Model development set

^b^Validation set

All studies were performed in compliance with the principles of the Declaration of Helsinki, the International Conference on Harmonization Good Clinical Practice (as defined in the International Conference on Harmonization E6 Guideline for Good Clinical Practice), and in accordance with applicable regulatory requirements. Furthermore, approval by the applicable ethics committees and the Federal Institute of Drugs and Medical Devices (BfArM, Bonn, Germany) were obtained prior to study conduct. All subjects gave written informed consent prior to participation in the studies and all data were anonymized prior to analysis.

Profiles were obtained following oral, i.v., and semi-simultaneous oral + i.v. administration of midazolam. Plasma concentrations were measured using validated ultra [30] or high [31] performance liquid chromatography–tandem mass spectrometry methods. The lower limits of quantification ranged from 50 fg/mL to 0.525 ng/mL for midazolam and from 250 fg/mL to 0.550 ng/mL for 1′-OH midazolam. Precision and accuracy of the assays were in accordance with health authority guidelines [32].

### Software

Models were developed using NONMEM 7.3 (ICON Development Solutions, Ellicott City, MD). The ADVAN6 subroutine was used and models were fit using the first-order conditional estimation with interaction (FOCE-I) method. NONMEM outputs and graphical diagnostics were processed in RStudio using R version 3.5.2 (R Foundation for Statistical Computing, Vienna, Austria) and Pearl-speaks-NONMEM (PsN; psn.sourceforge.net). Control stream code of the final model is provided in Online Resource 2.

### Population model development

Non-linear mixed-effects modeling was employed for the development of a parent-metabolite PopPK model for midazolam. The purpose of the model was to describe midazolam and 1′-OH midazolam concentrations in the presence of a DDI, with the intention of combining the model with limited sampling to reduce subject/patient burden while still accurately identifying presence of CYP3A DDIs. Model development was conducted in a stepwise manner, whereby separate base models were first developed for midazolam and for 1′-OH midazolam. The structurally defined base models were combined into one composite model, with a pre-systemic metabolic rate constant added and systemic fraction of midazolam metabolized fixed to 1. The pre-systemic metabolic rate constant was included in the composite model to account for the greater 1′-OH midazolam exposure seen following oral administration of midazolam compared to i.v. administration. Without this constant, oral concentrations were either systematically under-predicted or iv concentrations over-predicted during semi-simultaneous administration of midazolam. The systemic fraction metabolized was fixed to 1 due to the almost complete metabolism of midazolam via CYP3A and due to the limited contribution of minor metabolites to midazolam’s overall elimination.

For each analyte, 1-, 2-, and 3-compartment models were examined. A log-normal distribution was assumed for all pharmacokinetic parameters. Models were examined using a first-order absorption process (oral administration) with linear elimination. Given the rapid absorption time of midazolam, no lag time or transit compartments were assessed. Random effects for inter-individual variability (IIV) were modeled exponentially, due to the assumed log-normal distribution of the parameters. Initially, random effects for all parameters were examined and any effects smaller than 0.001 or leading to the model not minimising were removed from the model. Residual unexplained variance (RUV) was assessed using additive, proportional, and combined additive and proportional errors. Additionally, as more noise tends to be apparent in earlier sampling time points compared to later time points, residual error was further examined by splitting the chosen error term into early/late time points, referred to by Karlsson, Beal and Sheiner as a ‘Two-Step’ error model [33]. As suggested, the split was chosen such that the ‘early’ error term described the majority of the main absorption phase for both midazolam and 1′-OH midazolam, while the ‘late’ error term was meant to describe the disposition of the profiles.

During initial development, only one profile per subject was used in order to facilitate model development. For subjects with multiple constitutive activity profiles, these remaining profiles were used to build inter-occasion variability (IOV) into the final composite model. Inter-occasion variability was deemed important, given that the naturally occurring variation would otherwise result in additional noise for DDI interaction assessment. Once the appropriate structural and statistical models were ascertained, the covariates of age, weight, and sex were examined for influence on model parameters. Potential relationships with these covariates were first examined graphically; if correlations between any of the covariates and the model parameters were apparent, p-values were calculated and any covariate correlated with a p < 0.01 was added individually to the model. In order to be retained in the model, a drop in objective function value (OFV, based on the –2 log likelihood) of at least 3.84 points (corresponding to p < 0.05) was required, along with adequate precision in the estimation of the effect, the disappearance of a correlation between errors for the covariate and affected model parameter, along with a noticeable improvement in model fit based on model diagnostics. For age and weight, the following power models were used for assessment:

$$Q_{MP,i } = \left( {\theta_{QMP} \times \left( { \frac{weight,i}{{70\,kg}} } \right)^{\theta weight} } \right) \times e^{\eta i}$$

$$k_{met,i } = \left( {\theta_{kmet} \times \left( { \frac{age,i}{{26.5\,years}} } \right)^{\theta age} } \right) \times e^{\eta i}$$

where i refers to individual values, *θ*_QMP_ and *θ*_kmet_ refer to population values, *θ*_weight_ and *θ*_age_ refer to population level effects of weight and age, respectively, normalized to their approximate mean values (70 kg and 26.5 years) in the examined population, and *e*^*ηi*^ refers to the IIV.

Finally, covariance between parameters was examined graphically and p-values were calculated; apparent relationships were incorporated into the model when a drop in OFV of at least 3.84 points occurred.

### Model evaluation

Appropriateness of the models was evaluated based on physiological plausibility, the OFV, and goodness-of-fit plots. The chosen composite model was further assessed employing a visual predictive check (VPC) and sampling importance re-sampling (SIR); SIR analyses were conducted with 5 iterations, using 1000, 1000, 1000, 2000, and 2000 samples and 200, 400, 500, 1000, and 1000 re-samples, as suggested by Dosne et al. [34]. Finally, the additional treatment arms from the Fetzner et al. [27] study where midazolam was administered together with honey or artificial honey were used to assess model fit. As no interaction effect of honey consumption was observed, the treatment arms were considered suitable for external qualification of the composite model for constitutive CYP3A activity.

### Drug-drug interactions

The final composite midazolam/1′-OH midazolam model without CYP3A modulation was subsequently evaluated for its ability to describe CYP3A DDI presence through the inclusion of inhibition and induction treatment arms from the model development dataset. Model parameters were fixed and treatment was added as a covariate on F (bioavailability of midazolam), V_c_ (midazolam central compartment volume), k_met_ (pre-systemic rate of metabolism), Q_met_ (midazolam clearance via metabolism), and CL_met_ (metabolic clearance). Additional split error terms were added for inhibition, due to the delayed t_max_ compared to no CYP3A modulation. The resulting interaction parameters were then fixed and the model was run using the treatment arms where midazolam was administered following withdrawal of a potent inhibitor and inducer (induction was most prevalent) [28], following a single dose of efavirenz (weak induction/activation was present) [29], and following limited sampling of midazolam administered alone and together with potent CYP3A inhbitiors [18, 35–37], in order to test the robustness of the model. VPCs were conducted for each of the tested scenarios (constitutive, inhibited, and induced activity) and an additional SIR analysis was conducted for the final interaction model.

### Parameter cut-point assessment

Once the interaction model was found to provide an adequate description of the data from the CYP3A DDI arms, a subsequent evaluation using the R package ‘OneR’ was undertaken to determine the model predicted parameter that was best able to distinguish between treatment conditions (induction, inhibition, constitutive activity). The ‘OneR’ package applies a One Rule Machine Learning classification algorithm [38] (described as a 1-level decision tree) to determine parameter cut-points from numerous parameters which may be able to distinguish different outcomes (in this case, treatment categories). The percent of values assigned to the correct treatment, based on the cut-points, are output, along with a more detailed breakdown of the predicted versus actual category for the parameter with the highest accuracy.

## Results

### Study populations and pharmacokinetic data

The final dataset for examination of constitutive CYP3A activity contained 2371 and 2197 quantifiable observations from 99 healthy adult subjects for midazolam and 1′-OH midazolam, respectively. An additional 1077 and 961 quantifiable observations for midazolam and 1′-OH midazolam were available for further development of the model with DDI data. Subject information included sex, age, and weight. Subject characteristics for each of the studies and overall are given in Table 2.

**Table 2:** Population characteristics

	N	Age (years)		Sex	Weight (kg)	
		Mean	Range	M:F	Mean	Range
K119^a,b^	12	26.0	22–33	8:4	74.9	51–103
K155^a^	12	26.4	21–45	8:4	75.9	53.5–101
K169^a^	20	24.6	21–34	10:10	67.0	47–91
K257^a,b^	16	28.5	22–34	9:7	71.2	57–88
K380^a^	16	30.0	22–52	12:4	72.4	55.1–96.3
K194^a^	12	25.3	21–34	6:6	73.4	55–109
K345^a^	11	27.4	19–36	6:5	70.0	53.1–111
Total	99	26.9	19–52	59:40	71.7	47–111
K292^b^	16	32.7	22–49	12:4	73.4	61–92
K342^b^	12	29.8	19–50	8:4	74.7	50.0–94.1
K363^b^	18	33.7	24–50	7:11	67.9	52.8–88.9
Overall	145	28.6	19–52	86:59	71.7	47–111

^a^Model development set

^b^Validation set

### Composite midazolam model

**Fig. 1:**
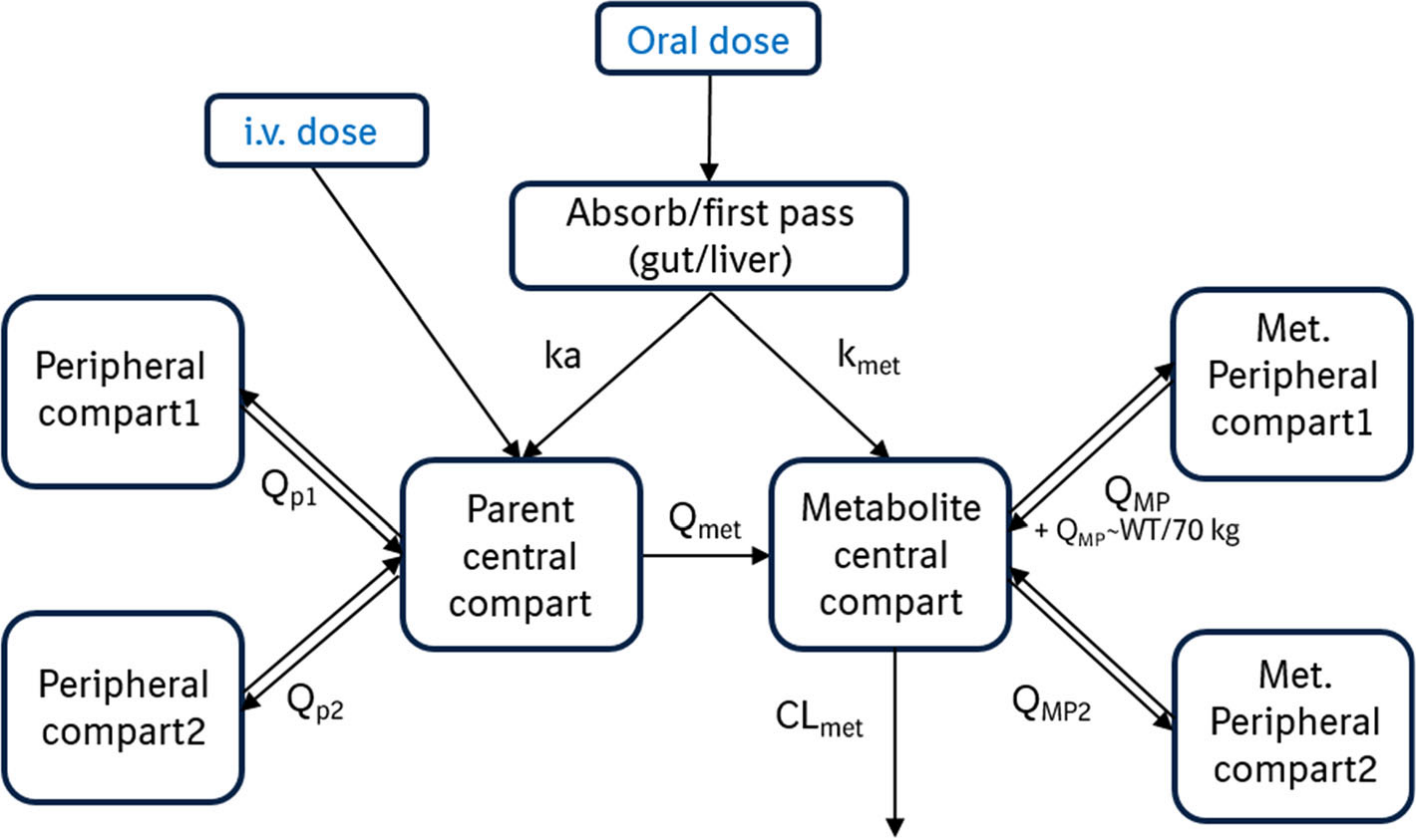
Adopted composite parent-metabolite midazolam model: 3-compartment model for each substance, first-order absorption and linear elimination; *k*_*met*_ pre-systemic metabolism rate, *ka* absorption rate constant; Q_p1_/Q_p2_/Q_MP_/Q_MP2_ = intercompartmental clearance, *Q*_*met*_ midazolam clearance via metabolism, *CL*_*met*_ metabolic clearance, *WT* weight

Twenty-nine midazolam measurements and 190 1′-OH midazolam concentrations were below the lower limit of quantification and were subsequently omitted from model development. A comparison of the 2-compartment models for both midazolam and 1′-OH midazolam with the respective 3-compartment models over time indicated that both analytes were best described using a 3-compartment drug disposition model with first-order absorption and linear elimination, as indicated by the comparably more even distribution of errors over time for the 3-compartment model (Online Resource 3, Fig. S1). The proportional error model, using an early/late cut-point at 0.5 h (the approximate t_max_), best fit the data, as indicated by a decrease in OFV and a narrower distribution of errors at early time points. No covariate relationships were apparent with any of the midazolam parameters, although effects were found for the 1′-OH midazolam parameters. Specifically, an effect was found between age and k_met_, such that an increase in age was related to decreased rate of pre-systemic metabolism, and between weight and inter-compartmental clearance for the first metabolic peripheral compartment, such that increased weight was related to increased inter-compartmental clearance. Addition of these covariate effects to the model resulted in decreased OFVs and a decrease in variability for inter-compartmental clearance. However, the addition of age did not reduce variability, nor did it appear to have any appreciable improvement in model fit based on visual inspection of diagnostic plots. Thus, only the effect between weight and inter-compartmental clearance was included in the composite model. A correlation between the EBEs for the volume of the midazolam central compartment and the volume of the metabolite’s central compartment was observed and included in the final model, with a significant improvement in model fit. The final composite model is displayed in Fig. 1.

PopPK parameter values, RSEs, shrinkage, and parameter uncertainty (as calculated using SIR) for the final model are presented in Table 3. Estimated bioavailability of midazolam was 27.6% and clearance was 24.1 L/h, which is congruent with values found in clinical studies [24]. The central volume for midazolam was estimated to be 19.5 L and the metabolic central volume was 175.6 L. These values suggest that both midazolam and 1′-OH midazolam are well-distributed in tissues throughout the body. The clearance estimate of 1′-OH midazolam was 196.8 L/h, showing a rapid clearance, similar to findings by others [39–42]. Thus, the population parameter values estimated from the model were deemed physiologically plausible.

**Table 3:** Population parameter values for the final adopted composite midazolam model and the model with interaction

Model parameters	Parameter name	Final model (N = 99 (4568 obs))		Interaction (N = 99 (6606 obs))			Parameter uncertainty:
		Value	RSE [%]	Shrinkage [%]	Value	RSE [%]	95% CI^a^ [lower, upper]
*Structural & covariate parameters*							
V_c_ [L]	MDZ central volume	19.5	8.08		FIXED		17.7, 21.4
V_p1_ [L]	MDZ peripheral 1 volume	41.0	7.92		FIXED		37.0, 45.8
V_p2_ [L]	MDZ peripheral 2 volume	23.8	4.75		FIXED		22.6, 25.1
Q_p1_ [h^−1^]	MDZ inter-compartment clearance 1	8.00	9.90		FIXED		7.10, 9.18
Q_p2_ [h^−1^]	MDZ inter-compartment clearance 2	46.1	8.26		FIXED		41.6, 51.8
ka parent [h^−1^]	Absorption rate constant	2.31	4.44		FIXED		2.20, 2.42
F	Combined bioavailability	0.276	3.35		FIXED		0.261, 0.291
k_met_ [h^−1^]	Pre-systemic metabolism rate	5.31	6.92		FIXED		4.83, 5.87
Q_met_ [L· h^−1^]	MDZ clearance via metabolism	24.1	2.00		FIXED		23.3, 25.1
V_met_ [L]	Metabolic central volume	175.6	9.69		FIXED		162, 193
V_MP_ [L]	Metabolic peripheral 1 volume	684.9	15.1		FIXED		604, 796
V_MP2_ [L]	Metabolic peripheral 2 volume	67.1	9.99		FIXED		56.4, 77.8
Q_MP_ [h^−1^]	Metabolic inter-compartment clearance 1	59.6	9.18		FIXED		51.9, 67.7
Q_MP,WT_ [h^−1^/70 kg]	Power of weight on Q_MP_	0.986	31.0		FIXED		0.409, 1.53
Q_MP2_ [h^−1^]	Metabolic inter-compartment clearance 2	127.4	11.0		FIXED		101, 155
CL_met_ [L· h^−1^]	Metabolic clearance	196.8	4.57		FIXED		186, 208
Early_MDZ,prop_	Early proportional error for MDZ (split: 0.5 h)	0.503	3.51		FIXED		0.471, 0.534
Late_MDZ,prop_	Late proportional error for MDZ (split: 0.5 h)	0.149	4.16		FIXED		0.143, 0.154
Early_1′-OHMDZ,prop_	Early proportional error for 1′-OH MDZ (split: 0.5 h)	0.556	3.32		FIXED		0.522, 0.590
Late_1′-OHMDZ,prop_	Late proportional error for 1′-OH MDZ (split: 0.5 h)	− 0.215	3.95		FIXED		− 0.224, − 0.208
*Inter-individual variability*							
ω^2^_Vc_	Variance of MDZ central volume	0.378	10.3	15.0	FIXED		0.311, 0.443
ω^2^_Vp1_	Variance of MDZ peripheral 1 volume	0.410	12.0	26.0	FIXED		0.339, 0.491
ω^2^_Qp1_	Variance of MDZ inter-compartment clearance 1	0.502	12.4	9.1	FIXED		0.426, 0.590
ω^2^_F_	Variance or bioavailability	0.234	13.4	19.4	FIXED		0.182, 0.289
ω^2^_kmet_	Variance of pre-systemic metabolism rate	0.369	9.76	12.9	FIXED		0.310, 0.445
ω^2^_Qmet_	Variance of MDZ clearance via metabolism	0.0934	39.3	48.6	FIXED		0.0339, 0.142
ω^2^_Vmet_	Variance of metabolic central volume	0.398	9.97	13.9	FIXED		0.326, 0.464
ω^2^_QMP_	Variance of metabolic inter-compartment clearance 1	0.485	12.2	20.9	FIXED		0.401, 0.585
ω^2^_CLmet_	Variance of metabolic clearance	0.133	31.7	56.1	FIXED		0.0602, 0.196
ω^2^_Vmet_ ~ ω^2^_Vc_	Covariance of MDZ and 1′-OH MDZ central volumes	0.731	13.2	–	FIXED		0.264, 0.392
*Inter-occasion variability*							
ω^2^_F_	Inter-occasion variability for bioavailability	0.162	14.0	40.3	FIXED		0.125, 0.202
ω^2^_Qmet_	Inter-occasion variability for MDZ clearance via metabolism	0.152	15.5	17.3	FIXED		0.127, 0.182
ω^2^_CLmet_	Inter-occasion variability for metabolic clearance	0.293	19.3	3.2	FIXED		0.251, 0.353
*Treatment effect*							
k_met,IND_ [h^−1^]	Induction effect on k_met_	–	–		12.4	30.6	9.20, 16.7
F_IND_ [%]	Induction effect on bioavailability	–	–		− 20.0	5.59	− 21.2, − 18.6
Q_met,IND_ [h^−1^]	Induction effect on midazolam clearance	–	–		38.0	20.5	30.5, 46.3
V_c,INH2_ [L]	Effect of irreversible inhibition on central volume	–	–		51.8	15.7	40.8, 65.2
k_met,INH2_ [h^−1^]	Irreversible inhibition effect on k_met_	–	–		− 5.24	0.362	− 5.26, − 5.20
F_INH1_ [%]	Reversible inhibition effect on bioavailability	–	–		37.9	6.87	33.4, 43.2
F_INH2_ [%]	Irreversible inhibition effect on bioavailability	–	–		134	14.2	114, 159
Q_met,INH1_ [h^−1^]	Reversible inhibition effect on midazolam clearance	–	–		− 16.3	3.77	− 16.9, − 15.7
Q_met,INH2_ [h^−1^]	Irreversible inhibition effect on midazolam clearance	–	–		− 12.7	9.99	− 14.0, − 11.2
CL_met,INH1_ [L· h^−1^]	Reversible inhibition effect on metabolic clearance	–	–		− 64.1	20.7	− 77.2, − 49.8
CL_met,INH2_ [L· h^−1^]	Irreversible inhibition effect on metabolic clearance	–	–		1117	11.2	901, 1387
Early_prop,INH_	Early proportional error for inhibition	–	–		0.483	4.49	0.456, 0.512
Late_prop,INH_	Later proportional error for inhibition	–	–		− 0.267	6.01	− 0.281, − 0.256

Relative standard errors for inter-individual variability are given on the approximate standard deviation scale (standard error/variance estimate)/2; *CI* confidence interval, *MDZ* midazolam, *obs* observations, *prop.* Proportional, *RSE* relative standard error

^a^Calculated using sampling importance resampling (SIR)

With regards to individual parameter values, random effects (IIV) were applied to central compartment volume (parent and metabolite), bioavailability, pre-systemic metabolic rate, clearance (parent and metabolite), inter-compartmental clearance and volume for peripheral compartment 1, and the inter-compartmental clearance for metabolic peripheral compartment 1. The application of IIV was based on the number of parameters where IIV could be precisely estimated, while still resulting in a stable model. Inter-occasion variability was added for the parameters thought to be most likely to vary with time, i.e. midazolam clearance, metabolic clearance, and bioavailability, and was estimated with good precision (all RSEs < 50%). Eta-shrinkage was generally below 30%, although for clearance (parent and metabolite), shrinkage was above 50%, suggesting that these estimates are likely biased.

**Fig. 2:**
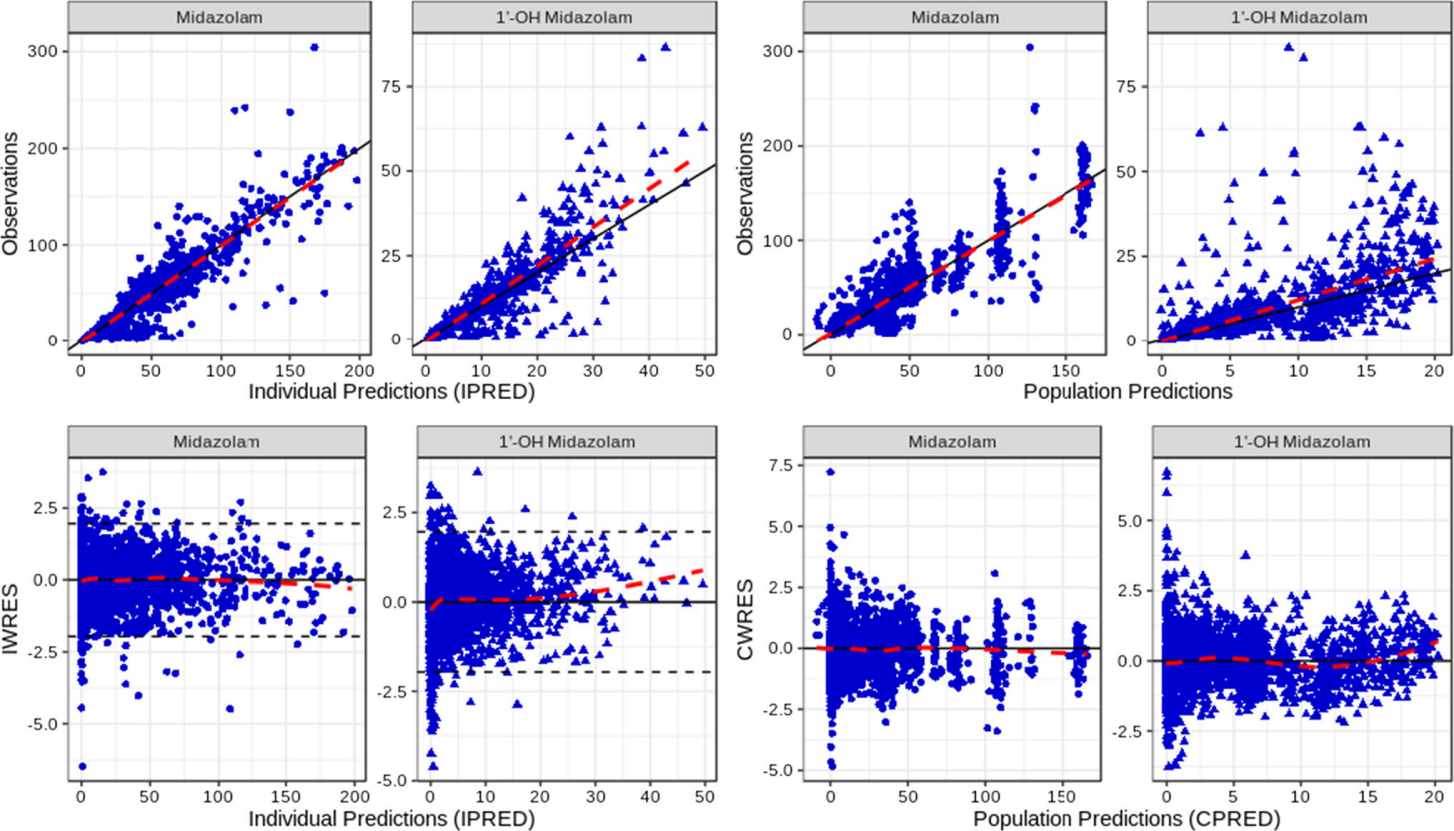
Goodness-of-fit plots for final composite model. *CPRED* conditional population predictions, *IPRED* individual predictions, *CWRES* conditional weighted residual error, *IWRES* individual weighted residual error. Points denote individual values; solid lines represent unity (top panels); the thick dashed line is a linear smoothing function (top panels) or a loess smoothing function (bottom panels); thin dashed lines represent ± 1.96 standard deviations (bottom left panels)

Goodness-of-fit plots for the final composite model are displayed in Fig. 2. Both individual and population predictions coincided well with the observed data, as noted by the smoothing line, which mostly overlapped with the line of unity. No trends were evident for individual or population predictions plotted against their respective weighted residual errors. Furthermore, the majority of individual weighted residual errors (other than for the lowest concentrations) fell within the range of ± 1.96, indicating good model fit. The final model was also found to provide a good fit to the midazolam + honey and midazolam + artificial honey treatment arms (Online Resource 4, Fig. S2 base conditions).

SIR diagnostic plots indicated convergence was achieved. RSEs were generally congruent with those obtained from the covariance-matrix in NONMEM, with marginally smaller RSEs obtained from the SIR analyses for parameter estimates and larger RSEs for variability estimates. Plots showing the convergence diagnostics (Fig. S3), as well as the 95% CIs over the parameters at different iterations (Fig. S4) are presented in Online Resource 5, while numerical results are presented in Table 3. The 95% CIs for key parameters (V_c_, ka, k_met_, F, Q_met_, CL_met_) and for IIV and IOV were generally relatively narrow. With regards to the covariate effect of weight, the 95% CI was quite wide, showing high parameter uncertainty, likely due to the limited range of weights incorporated in the analysis and the still relatively small effect explained by the addition of weight. Asymmetries in CIs were noted for the majority of variance parameters, while the volume of the metabolite peripheral 1 compartment also displayed an asymmetric 95% CI.

### Effect of perpetrators: interaction model

**Fig. 3:**
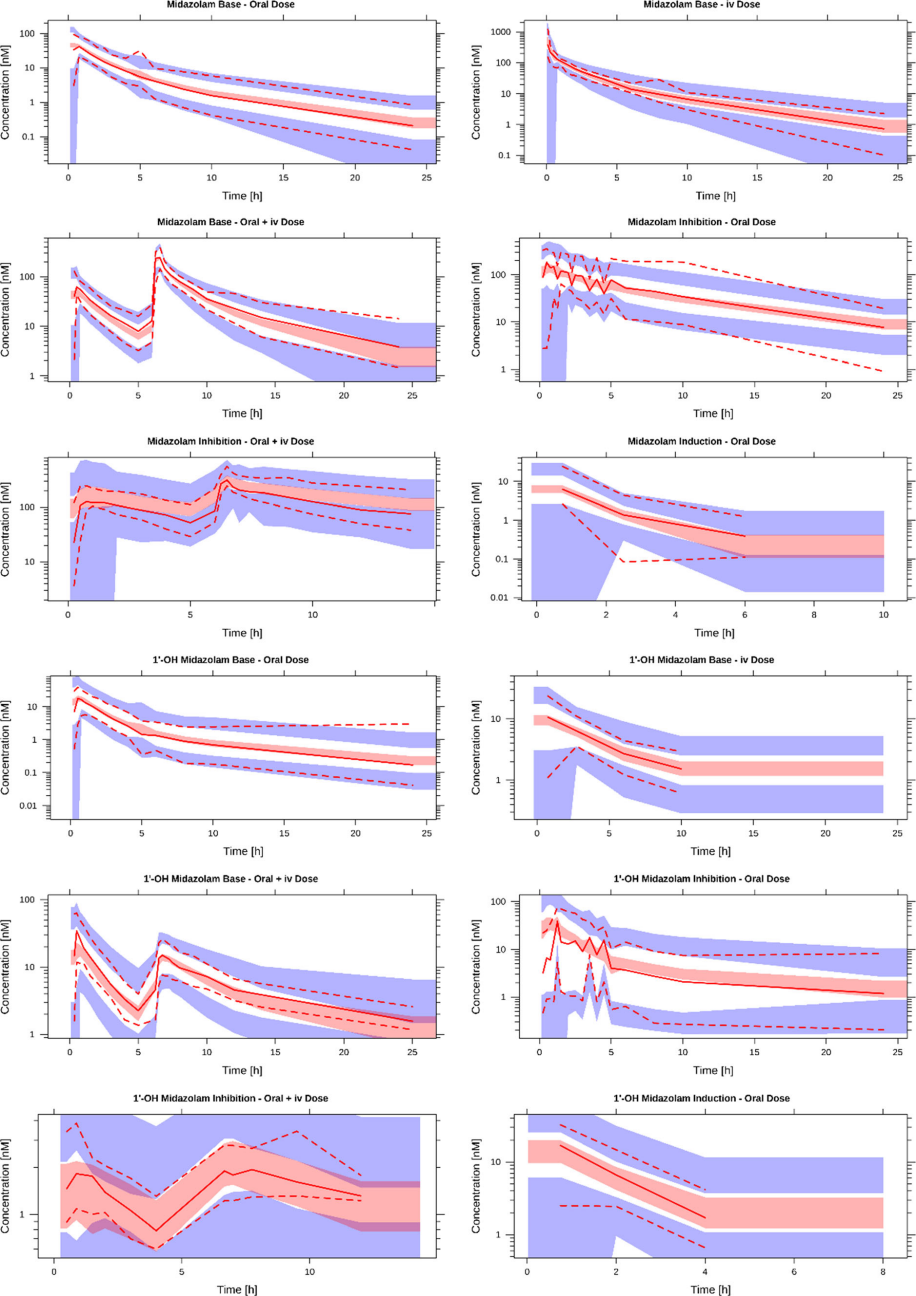
Visual predictive checks (1000 simulations) for the final interaction model. *nM* nanomolar, *h* hours. Solid lines depict the observed median concentrations; dotted lines depict the observed 97.5th and 2.5th percentiles. The middle shaded area pertains to the 90% confidence interval for the predicted medians, while the outer shaded areas pertain to the 90% confidence interval for the 97.5th and 2.5th percentile predictions. Data are normalized to a midazolam dose of 4 mg (semi-log scale)

Visual inspection of concentration–time profiles of midazolam and 1′-OH midazolam indicated differential effects of reversible (e.g. by ketoconazole or voriconazole) and irreversible (e.g. by ritonavir) inhibition. Thus, inhibition was split into two components: one for reversible and one for irreversible inhibition. Effects that could not be well estimated were removed from the model, resulting in the removal of the reversible inhibition effect on pre-systemic rate of metabolism (RSE > 80%). The addition of an irreversible inhibition effect on central volume of distribution was required to adequately capture midazolam disposition. Due to differences in time to t_max_, the proportional error term with an early/late split at 1.5 h (approximate t_max_) for inhibition provided a better fit to the data than the original split at 0.5 h. Resulting values, RSEs, and SIR-based parameter uncertainty are provided in Table 3. The resulting model was able to adequately describe both midazolam and 1′-OH midazolam concentrations following administration with a potent CYP3A inhibitor (ketoconazole, voriconazole, or ritonavir) or inducer (efavirenz) (Fig. 3).

As seen in Table 3, the inhibition effects resulted in a 0.379 increase in bioavailability, a 16.3 L/h decrease in midazolam clearance, and a 64.1 L/h reduction in metabolic clearance for reversible inhibition, while for irreversible inhibition, effects were generally more pronounced, with a 5.24 /h reduction in pre-systemic metabolism rate, a 1.34 increase in bioavailability, a 51.8 L increase in midazolam central volume, a 12.7 L/h decrease in midazolam clearance, and a 1117 L/h higher metabolic clearance. The decreased rate of pre-systemic metabolism and increased bioavailability, combined with decreased midazolam clearance due to CYP3A inhibition resulted in the expected higher midazolam exposures. A differential effect of reversible and irreversible inhibition on metabolic clearance resulted in a considerably reduced exposure to 1′-OH midazolam for irreversible inhibition due to the increased clearance, while the decreased clearance of metabolite following reversible inhibition resulted in more 1′-OH midazolam still present in systemic circulation.

The expected opposite effect following induction of CYP3A was evident through the 12.4 L/h increased rate of pre-systemic metabolism, a 0.200 decrease in bioavailability, and a 38.0 L/h increase in midazolam clearance. These changes resulting from the model are plausible, given the increased metabolic activity and lead to the expected decreased midazolam exposure and increased 1′-OH midazolam exposure.

With regards to model evaluation, SIR analyses indicated convergence was achieved within the 5 iterations. RSEs were generally congruent with those obtained from the covariance-matrix in NONMEM, albeit with smaller RSEs obtained from the SIR analyses. Plots showing convergence diagnostics (Fig. S5), as well as the 95% CIs over the parameters at different iterations (Fig. S6) are presented in Online Resource 5, while numerical results are presented in Table 3. CIs for the treatment effects were generally wider than those seen for the composite model parameters, particularly for metabolic clearance and changes in bioavailability following irreversible inhibition. Asymmetries were noted for the irreversible inhibition effect on midazolam central volume and for midazolam clearance following induction.

VPCs of the model for the external validation data are provided in Online Resource 4 (Fig. S2). Midazolam and 1′-OH midazolam concentrations were adequately described for constitutive activity and inhibition, although the 2.5th percentile for midazolam following inhibition was over-predicted and the median for 1′-OH midazolam was under-predicted. For induction, midazolam concentrations were under-predicted, while 1′-OH midazolam concentrations were better captured by the model, particularly following oral administration.

### Cut-points

**Fig. 4:**
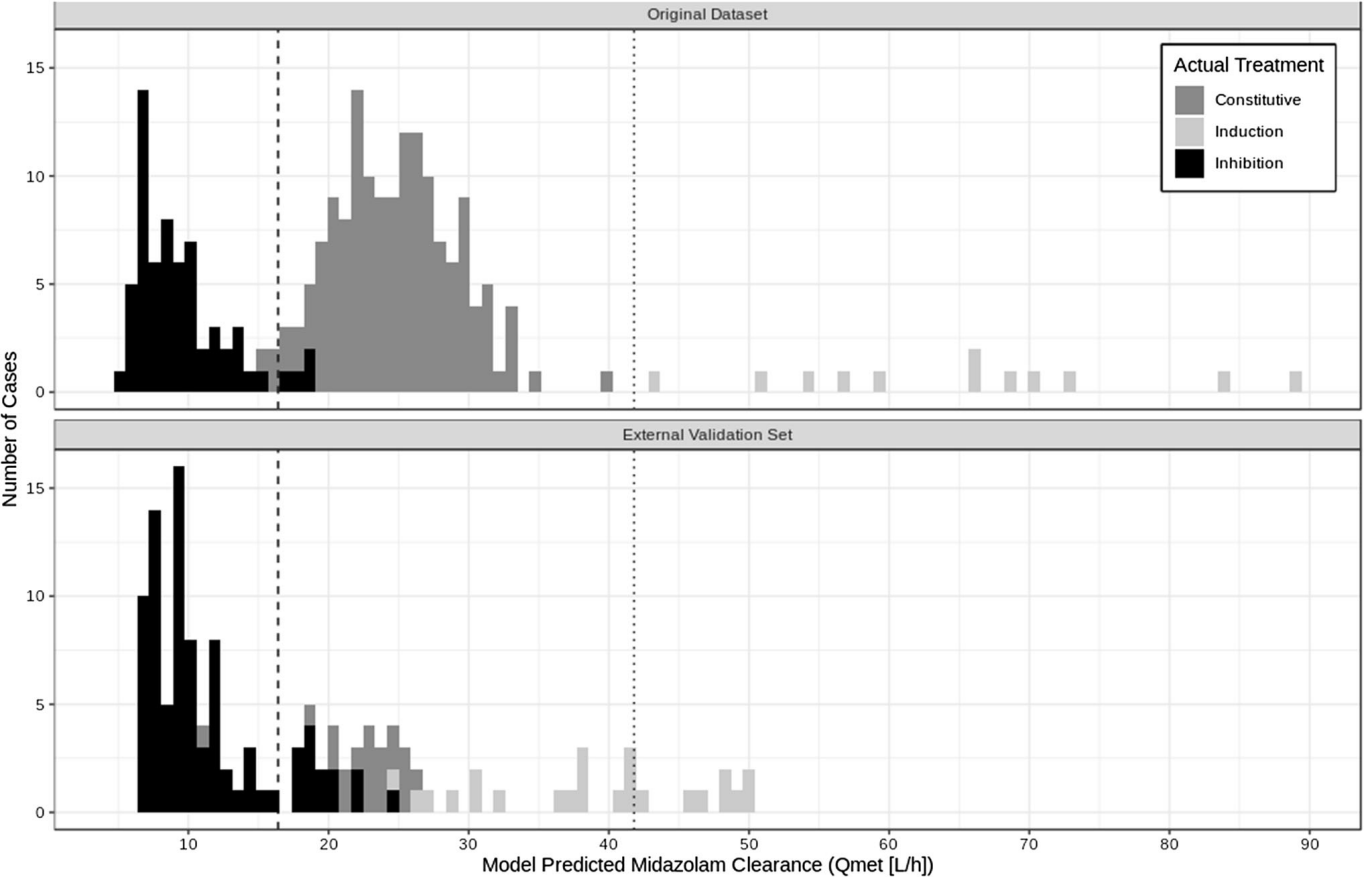
Treatment arm distribution based on midazolam clearance (Q_met_). The dashed line represents the cut-point at 16.4 L/h, separating predictions for inhibition (all below 16.4) and no modulation, while the dotted line represents the cut-point at 41.8 L/h separating no modulation from induction (everything above 41.8). Shading represents the actual treatment condition, with black showing inhibition, dark grey showing constitutive activity, and light grey showing induction

Using model estimated parameters, an assessment of potential cut-points for identifying the different CYP3A modulation categories (constitutive/no modulation, inhibition, and induction) was conducted based on the outcomes from the development set of the interaction model. The estimated model parameter with the highest accuracy in identifying modulation categories was midazolam clearance via metabolism (i.e. Q_met_). Using the cut-points 4.82–16.4 L/h (inhibition), 16.4–41.8 L/h (no modulation), and 41.8–88.9 L/h (induction), 97.3% of the treatments arms were correctly categorized, as displayed in Table 4 and Fig. 4. Of the misclassified arms, 4/63 (6.35%) of inhibition cases were classified as having no modulation and 2/148 (1.35%) cases with no modulation of CYP3A were classified as inhibition. All of the induction cases were correctly identified. For the validation set, 76.9% of the cases were correctly classified. The majority of misclassifications were in the induction treatment group, with 15/24 (62.5%) cases classified as no modulation. Eighty-three percent of inhibition cases were correctly categorized, with the remainder classified as no modulation (14/86); 1/20 (5.0%) no modulation cases were classified as inhibition, with all others correctly classified. Removing the weak induction arm (single dose of efavirenz) increased the accuracy to 83.9%, with the number of misclassified induction cases reduced to 4/12 (33.3%). Thus, for the combined datasets, 165/168 no modulation cases (specificity = 98.2%), 131/149 inhibition DDIs (sensitivity = 87.9%), and 21/36 induction DDIs (sensitivity = 58.3%) were correctly identified. Removal of weak induction resulted in a sensitivity of 83.3% for potent induction.

**Table 4:** Predicted versus actual treatment category based on midazolam clearance (Q_met_) cut-points

Predicted category and midazolam clearance cut-point	Inhibition	No modulation	Induction
	(4.82–16.4 L/h)	(16.4–41.8 L/h)	(41.8–88.9 L/h)
*Actual treatment—original dataset*			
Inhibition [N (%)]	59 (93.7)	4 (6.35)	0 (0)
No modulation [N (%)]	2 (1.35)	146 (98.6)	0 (0)
Induction [N (%)]	0 (0)	0 (0)	12 (100)
*Actual treatment—external validation set*			
Inhibition [N (%)]	72 (83.7)	14 (16.3)	0 (0)
No modulation [N (%)]	1 (5.00)	19 (95.0)	0 (0)
Induction [N (%)]	0 (0)	15 (62.5)	9 (37.5)
*Actual treatment—external validation set, no weak induction*			
Inhibition [N (%)]	72 (83.7)	14 (16.3)	0 (0)
No modulation [N (%)]	1 (5.00)	19 (95.0)	0 (0)
Induction [N (%)]	0 (0)	4 (33.3)	8 (66.7)
*Actual treatment—both sets*			
Inhibition [N (%)]	131 (87.9)	18 (12.1)	0 (0)
No modulation [N (%)]	3 (1.79)	165 (98.2)	0 (0)
Induction [N (%)]	0 (0)	15 (41.7)	21 (58.3)
*Actual treatment—both sets, no weak induction*			
Inhibition [N (%)]	131 (87.9)	18 (12.1)	0 (0)
No modulation [N (%)]	3 (1.79)	165 (98.2)	0 (0)
Induction [N (%)]	0 (0)	4 (16.7)	20 (83.3)

## Discussion

The aim of the present model was to describe the pharmacokinetics of midazolam and 1′-OH midazolam during constitutive, inhibited, and induced CYP3A activity in healthy adult subjects for future use in combination with limited sampling strategies. Numerous midazolam PopPK models have been developed previously [39–41, 43–45], although none have assessed midazolam during CYP3A inhibition or induction; this has generally been left to physiologically-based pharmacokinetic [PBPK] models, which are more labour-intensive to develop and require greater amounts of information for both the perpetrator and victim than may be available in early drug development. The final composite model was best described with 3-compartments, first-order absorption and linear elimination for both analytes, with a two-step proportional residual error. Treatment effects on F, V_c_, Q_met_, k_met_ and CL_met_, as well as a proportional error term for the delayed t_max_ by inhibition allowed for adequate description of the midazolam and 1′-OH midazolam concentrations during inhibition and induction. As an exploratory objective, potential parameter cut-points were examined for identifying CYP3A activity conditions, for potential use when only a single midazolam profile is available.

To account for the metabolic activity which midazolam undergoes, certain parameters were required in the composite and interaction models. Specifically, the inclusion of pre-systemic metabolism rate was found to be necessary to properly capture the increased 1′-OH midazolam concentrations following oral administration of midazolam compared to iv administration. Given that it is known that midazolam undergoes extensive first-pass metabolism, this addition was deemed appropriate. Furthermore, it was necessary to fix the systemic fraction metabolized to increase model stability. Given the almost complete metabolism of midazolam and the inability to detect the fractions of each metabolite (due to only having info on 1′-OH midazolam), a fraction metabolized of 1 was chosen. A sensitivity analysis fixing fraction metabolized to 0.8 and to 0.6 (approximate fraction of midazolam recovered as 1′-OH midazolam glucuronide in urine [24, 25]) revealed a minimal impact on parameter estimates, although RSEs were considerably higher for the lower fractions.

Composite model estimates for midazolam were in-line with those found by others [44, 45] and with CL and F values found in clinical studies [24], showing physiological plausibility. Proportional error was found to be comparable with the estimates from the model by Yang et al. [44], who also applied a two-step approach, supporting the use of this method for midazolam. Estimates of 1′-OH midazolam CL_met_ were also in accordance with previous models [39–41]. The examination of sex, age, and weight as potential covariates revealed relationships between age and k_met_, as well as between weight and metabolite inter-compartmental clearance. However, only weight appeared to have any appreciable impact in reducing unexplained varability. Sex was not found to be a significant covariate. Thus, the final composite model only included weight as a covariate.

Applying treatment effects to the final composite model for midazolam clearance, central compartment volume, pre-systemic rate of metabolism, bioavailability, and metabolic clearance resulted in a good description of the data following CYP3A inhibition and induction with potent perpetrators, although 1′-OH midazolam concentrations were generally less well-described. Multiple factors may help explain this. First, inhibition via both reversible and irreversible inhibition was included in the model; as these have different mechanisms of action, they would not necessarily be expected to impact exposure in the same way. Furthermore, one of the included reversible inhibitors, voriconazole, has been noted by Li et al. [46] to also display time-dependent inhibition, thus encompassing characteristics of both reversible and irreversible inhibitors. In support of the differing impact of reversible and irreversible inhibitors, inclusion of split terms significantly improved the model fit for the data. Given the differential impact of the two mechanisms of action, the mixed inhibitory characteristics of voriconazole would have been harder to capture in the current model. Thus, more data examining each of the inhibitory conditions (reversible, mixed reversible/irreversible, and irreversible) would help to further tease out the impact of the different types of inhibition on 1′-OH midazolam exposure.

Further potential reasons that CYP3A activity may not have been as well accounted for with 1′-OH midazolam is the already present inter-individual differences in CYP3A activity, as well as the fact that the metabolite undergoes further metabolism [24, 25]. The inter-individual differences may be particularly evident during CYP3A modulation, as inhibition would have a greater impact on those whose CYP3A activity is higher at baseline, while induction may have a greater impact for those with lower levels of CYP3A activity initially. With regards to 1′-OH midazolam’s metabolism, a perpetrator may not only be a perpetrator of CYP3A, but also of phase II metabolic enzymes, thereby influencing further metabolism (and, thus, clearance) of this metabolite. Frechen et al. [26] noted that voriconazole weakly inhibits further metabolism of 1′-OH midazolam, providing evidence of such additional interactions. This hypothesis is further supported by the decreased metabolic clearance noted for reversible inhibition in the present model. The combination of already high inter-individual variability in CYP3A activity, combined with the potential for differences regarding impact on further metabolism would introduce a greater level of variability into the model compared to that present for midazolam.

When the composite interaction model was evaluated using the external validation set, midazolam concentrations were generally well-described for constitutive and inhibitory conditions (although the 2.5th percentile for inhibition was over-predicted), but were under-predicted for induction. The under-prediction likely stems from the fact that the external dataset included treatment arms which showed strong induction effects (following withdrawal of a potent inducer) and weak induction effects (following a single dose of efavirenz). Thus, future models may wish to build in a potency factor using known inhibitors and inducers of various potencies to account for the different modulatory effects. With regards to 1′-OH midazolam concentrations, baseline was generally well-described, as was the range of inhibition concentrations, although the median for inhibition was under-predicted. Induction effects were less well-described, particularly following a semi-simulataneous iv dose, although the median and 97.5th percentiles following oral dosing were satisfactorily captured. Given the satisfactory description of the data seen in the VPCs for the external dataset for all but midazolam induction, the model shows adequate external validity. Furthermore, both the non-modulatory conditions and the inhibition conditions included limited sampling datasets, suggesting the model is appropriate for use with sparse sampling, at least for these two conditions.

Although some under-estimation of the model was apparent for induction, the model was still deemed able to serve its desired purpose. Specifically, the desired application of this model is for detecting the true presence of DDIs in vivo early in drug development. Thus, the under-prediction of concentrations (i.e. the lower concentrations of midazolam predicted by the model) following induction would still allow for such an assessment, although the extent of the effect may be overestimated. Should the presence of a DDI be noted, then time and resources may be further invested for the development of a PBPK model, which would be able to provide a more accurate description of the extent of DDI based on the test compound’s concentrations and, thus, also be used for describing DDI scenarios using different dosing schemes. As the development of PBPK models is very time and labour-intensive, it is desirable to only embark upon development of such a model once it is clear that one may be needed. Thus, this PopPK model may be seen as a complementary initial tool for DDI assessment which can give earlier information regarding the need for excluding CYP3A substrates in Phase II studies, as well as informing on the need for PBPK model development. In this way, considerable time and resources can be saved while still obtaining valuable information.

The exploratory evaluation of potential cut-points using the model development dataset indicated clear differences in estimated midazolam clearance for all three conditions tested (constitutive, inhibition, induction). Thus, model estimated clearance could be split such that almost 100% of cases were correctly identified following administration without CYP3A modulation, (16.4–41.8 L/h), with potent inhibitors (4.82–16.4 L/h), and with a potent inducer (41.8–88.9 L/h). However, when applied to the external validation set, induction was less well categorized. The majority of the misclassified induction effects were for the treatment arms following a single dose of efavirenz and represented weak induction effects. As model estimated clearance following moderate or weak modulation would result in greater overlap between conditions, weak modulation may not be as well detected and further research examing the influence of different potencies of CYP3A modulators is warranted. Given the clear differences between clearance values for the rest of the data, however, at least potent and potentially moderate modulation are likely to be correctly identified using the cut-offs here. Therefore, should these cut-points also prove to be valid for patient populations, they may provide valuable information in clinical practice when assessing CYP3A DDIs where baseline measures are not always available.

## Limitations

Several limitations were present within the developed model. In particular, the amount of data for induction was relatively limited (only 1 study with full profiles, consisting of only 12 subjects). As such, although the individual predictions were generally adequate, the fit for the overall population would likely have been better with the inclusion of more data during model development. Furthermore, only potent modulators were seen to be well-described, suggesting further data and adjustments may be needed for moderate and or weak modulation.

Another limitation of the current model is the inability to describe the time-course of inhibition. In models by Frechen et al. [26] and Li et al. [46], it was shown that there is a variable time-course to the inhibition by voriconazole, thus, the ability to capture this time-course would likely result in more accurate DDI predictions and provide a better estimation of different levels of DDI modulation. However, as stated previously, the main purpose of the current model is to be able to get a more accurate assessment of presence of in vivo DDIs early in drug development while limiting required resources and inconvenience for subjects/patients, thereby informing steps which may be necessary in the subsequent development program (e.g. need for a PBPK model).

Finally, with regards to covariate analyses, the age and weight ranges included in the present study were still relatively small (19–52 years and 47–111 kg), so results from the model do not necessarily extend to pediatric or elderly populations, or to obese individuals, thus, additional research examining the current model with different populations, including patient populations, would be needed.

## Conclusion

The developed PopPK model was able to describe midazolam and 1′-OH midazolam exposure in healthy adults during constitutive, inhibited, and induced CYP3A activity. Furthermore, the model was able to describe such activity following limited sampling alone or in the presence of an inhibitor, suggesting it can be combined with sparse sampling in the future. The obtained cut-points for clearance may be useful indicators of interaction presence when only a single profile is available, suggesting a particularly convenient application for clinical practice if also confirmed as relevant for patient populations.

## Electronic supplementary material

Below is the link to the electronic supplementary material.

Supplementary file1 (PDF 406 kb) Table S1 Study DesignsAdopted Composite Model Control Stream with Interaction

Supplementary file2 (PDF 340 kb) Fig. S1 Comparison of 2- and 3-Compartment Model Error Distributions

Supplementary file3 (PDF 594 kb) Fig. S2 Composite Midazolam Interaction Model VPC – External Validation

Supplementary file4 (PDF 661 kb) Figs. S3-S6 Sampling Importance Resampling of Composite and Interaction Models

Supplementary file5 (PDF 1369 kb)
