# Supplementary material for: Composite midazolam and 1′-OH midazolam population pharmacokinetic model for constitutive, inhibited and induced CYP3A activity
Source: J Pharmacokinet Pharmacodyn. 2020 Aug 8;47(6):527–42. doi: 10.1007/s10928-020-09704-1 (PMC7652802; doi:10.1007/s10928-020-09704-1)
Supplement: Supplementary file 1 — Supplementary file1 (PDF 406 kb) Table S1 Study DesignsAdopted Composite Model Control Stream with Interaction [file 10928_2020_9704_MOESM1_ESM.pdf]

ORIGINAL PAPER

Composite midazolam and 1'-OH midazolam population pharmacokinetic model for constitutive, inhibited and induced CYP3A activity

Sabrina T. Wiebe[1,2], Andreas D. Meid[1], Gerd Mikus[1]

[1]Department of Clinical Pharmacology and Pharmacoepidemiology, University of Heidelberg, Im Neuenheimer Feld 410, 69120 Heidelberg, Germany

[2]Boehringer Ingelheim Pharma GmbH & Co. KG, Birkendorfer Str. 65, 88397 Biberach an der Riss, Germany

Correspondence: Professor Gerd Mikus MD, Department of Clinical Pharmacology and Pharmacoepidemiology, University of Heidelberg, Im Neuenheimer Feld 410, 69120 Heidelberg, Germany; Tel.: +4962 2156 8740; Fax: +4962 2156 4642; E-mail: gerd.mikus@med.uni-heidelberg.de

Table S1 Study Designs for Composite and Interaction Model Datasets

Study	Design and model inclusion	Reference
K119[a,b]	This was an open-label, fixed sequence study, with 4 assessment periods. Pharmacokinetics of 12 healthy subjects was assessed for midazolam given orally (4 mg) followed by iv administration (2 mg) 6 hours later during the following conditions:<br><br>1) baseline;<br>2) following a single dose of either St. John's wort (6 subjects) or ritonavir (6 subjects);<br>3) following 14 days of co-administration of St. John's wort (300 mg tid) and ritonavir (300 mg bid); and<br>4) following cessation of St. John's wort and ritonavir.<br><br>For model development, conditions 1 and 3 were used, while the cessation period was used in the external validation set.	[1]
K155[a]	This was an open-label, fixed sequence study with 2 assessment periods. Pharmacokinetics of 12 healthy subjects was assessed for a single-dose of midazolam given orally (3 mg) during the following conditions:<br><br>1) baseline;<br>2) following 14 days of efavirenz (400 mg qd) administration.<br><br>Both conditions were used for model development.	[2]
K169[a]	This was a randomized, single-blind, parallel group study, with 4 assessment periods. Pharmacokinetics of 20 healthy subjects was assessed for midazolam given orally (4 mg) followed by iv administration (2 mg) 6 hours later during the following conditions:<br><br>1) baseline;<br>2) following 10 days honey (2x20 g/day; 10 subjects) or artificial honey (2x20 g/day; 10 subjects);<br>3) 1 day following cessation of (artificial) honey consumption;<br>4) 6 days following cessation of artificial honey consumption.<br><br>Baseline was used for model development, while all other conditions were used for model evaluation.	[3]

K257[a]	This was a randomized, open-label, fixed sequence, parallel group study, with 10 assessment periods. Pharmacokinetics of 16 healthy subjects was assessed for a single-dose of midazolam given orally (3 mg) during the following conditions:<br><br>1) baseline;<br>2) following 1, 2, 3, 5, 8, and 9 days of voriconazole (day 1: 400 mg bid orally, days 2–9: 200 mg bid orally, with only a morning dose on day 9; 8 subjects) or ritonavir (300 mg bid orally, with only a morning dose on day 9; 8 subjects);<br>3) 1, 2, and 3 days following cessation of voriconazole or ritonavir administration.<br><br>Baseline was used for model development, as were days 1, 2, 3, and 9 of voriconazole treatment and days 2 and 3 of ritonavir treatment (due to time-dependent inhibition, day 1 was not used and due to a potential autoinductive effect of ritonavir [4], day 9 was not used).	[5]
K380[a]	This was an open-label, fixed sequence, two-arm study, with 4 different midazolam doses examined. Sixteen healthy subjects received midazolam doses orally (3 µg and 3 mg) and intravenously over 5 min (1 µg and 1 mg), followed by 4 randomized treatments of midazolam (1 µg iv or 3 µg po) + voriconazole (50 mg iv and po, and 400 mg iv and po). The 4 combinations, given in 2 different randomisation arms (always starting with 50 mg voriconazole), were:<br><br>1) 50 mg po voriconazole + 1 µg iv midazolam<br>2) 50 mg iv voriconazole + 3 µg po midazolam<br>3) 400 mg po voriconazole + 1 µg iv midazolam<br>4) 400 mg iv voriconazole + 3 µg po midazolam<br><br>Washout periods between initial midazolam doses were at least 72 h and between all subsequent conditions were at least 7 days.<br><br>The 1 µg dose group was not used for model development, due to the large portion of LLOQ values for 1’-OH midazolam, as well as a greater model instability when including this dose group. Additionally, as the 50 mg voriconazole condition did not consistently result in inhibition, only the 400 mg dose of voriconazole was examined in the model.	[6,7]

K194[a]	This was an open-label, fixed sequence study, with 6 assessment periods. Pharmacokinetics of 12 healthy subjects was assessed for midazolam given orally (4 mg) followed by iv administration (2 mg) 6 hours later during the following conditions:<br><br>1) baseline;<br>2) 1, 6, 11, 16, and 22 days following single dose administration of efavirenz (400 mg, administered 12 hours before initial midazolam dose).<br><br>The baseline condition was used for model development, while days 1 and 6 following efavirenz administration, when activation effects were greatest, were included in the external validation set.	[8]
K345[a]	This was an open-label, randomized, fixed sequence, two group combination single ascending dose and drug-drug interaction study. Four different doses of midazolam (100 ng, 1 µg, 100 µg, and 1 mg [6 subjects] or 300 ng, 3 µg, 30 µg, and 3 mg [6 subjects]) were examined in the ascending dose portion, while 2 doses (100 ng [6 subjects] or 300 ng [6 subjects] and 1 mg or 3 mg, respectively) were examined in combination with ketoconazole (400 mg qd for 15 days). Twelve healthy subjects received midazolam doses orally, with pharmacokinetic assessments at the following occasions:<br><br>1) following each individual dose in the ascending dose portion;<br>2) following day 2 of ketoconazole administration for both the nanogram and milligram doses;<br>3) following day 8 of ketoconazole administration for both the nanogram and milligram doses.<br><br>Nanogram doses and 1 µg were excluded from model development and evaluation, due to the high proportion of LLOQ values for 1'-OH midazolam, as well as instability of the model for these low doses.	[9]
K292[b]	This was an open-label, randomised, crossover study, with three treatment periods and limited sampling. Pharmacokinetics of 16 healthy subjects was assessed for a single-dose of midazolam given orally (3 mg) during the following conditions:<br><br>1) baseline;<br>2) midazolam + fentanyl (5 µg/kg);<br>3) midazolam + fentanyl (5 µg/kg) + ketoconazole (200 mg bid; midazolam given on 2nd day of administration);	[10]

	Washout between fentanyl treatment periods was at least 6 days. The baseline condition and midazolam + fentanyl + ketoconazole were both included as part of the external validation set.	
K342[b]	This was an open-label, randomised, fixed sequence, two group study. Pharmacokinetics of 12 healthy subjects was assessed for a single-dose of midazolam given orally (3 mg) during the following conditions:<br><br>1) baseline;<br>2) following 4 increasing doses of ritonavir (0.1, 1, 10, 100 mg [6 subjects] or 0.3, 3, 30, 300 mg [6 subjects]); treatments were separated by washout periods of at least 2-7 days.<br><br>The baseline condition and the condition with the highest ritonavir dose were both included in the external validation set.	[11]
K363[b]	This was a two-part study; only the first part was examined here. This first part was open-label and fixed sequence, with two treatment conditions. Pharmacokinetics of 18 healthy subjects was assessed for a single-dose of midazolam (100 µg) given orally using limited sampling during the following conditions:<br><br>1) baseline;<br>2) following administration of a low dose of ritonavir (40 mg).<br><br>Both conditions were included in the external validation set.	[12]

[a]Model development set.
[b]Validation set
iv = intravenous; po = *per os* (oral); qd = *quaque die* (once daily); bid = *bis in die* (twice daily); tid = *ter in die* (three times daily)

References

1. Hafner V, Jäger M, Matthée AK, Ding R, Burhenne J, Haefeli W, Mikus G (2010) Effect of simultaneous induction and inhibition of CYP3A by St John's Wort and ritonavir on CYP3A activity. Clinical Pharmacology & Therapeutics 87 (2):191-196

2. Heinrich T, Röder C, Gehrig A, Haefeli W, Mikus G (2007) Impact of long-term (14 days) efavirenz administration on CYP3A4 activity. Basic & Clinical Pharmacology & Toxicology 101 (5):373-374

3. Fetzner L, Burhenne J, Weiss J, Völker M, Unger M, Mikus G, Haefeli WE (2011) Daily honey consumption does not change CYP3A activity in humans. The Journal of Clinical Pharmacology 51 (8):1223-1232

4. Hsu A, Granneman GR, Witt G, Locke C, Denissen J, Molla A, Valdes J, Smith J, Erdman K, Lyons N (1997) Multiple-dose pharmacokinetics of ritonavir in human immunodeficiency virus-infected subjects. Antimicrobial agents and chemotherapy 41 (5):898-905

5. Katzenmaier S, Markert C, Riedel K, Burhenne J, Haefeli W, Mikus G (2011) Determining the time course of CYP3A inhibition by potent reversible and irreversible CYP3A inhibitors using a limited sampling strategy. Clinical Pharmacology & Therapeutics 90 (5):666-673

6. Hohmann N, Kocheise F, Carls A, Burhenne J, Weiss J, Haefeli WE, Mikus G (2016) Dose-dependent bioavailability and CYP3A inhibition contribute to non-linear pharmacokinetics of voriconazole. Clinical pharmacokinetics 55 (12):1535-1545

7. Hohmann N, Kocheise F, Carls A, Burhenne J, Haefeli WE, Mikus G (2015) Midazolam microdose to determine systemic and pre-systemic metabolic CYP3A activity in humans. British journal of clinical pharmacology 79 (2):278-285

8. Mikus G, Heinrich T, Bödigheimer J, Röder C, Matthee AK, Weiss J, Burhenne J, Haefeli WE (2017) Semisimultaneous midazolam administration to evaluate the time course of CYP3A activation by a single oral dose of efavirenz. The Journal of Clinical Pharmacology 57 (7):899-905

9. Halama B, Hohmann N, Burhenne J, Weiss J, Mikus G, Haefeli WE (2013) A nanogram dose of the CYP3A probe substrate midazolam to evaluate drug interactions. Clin Pharmacol Ther 93 (6):564-571. doi:10.1038/clpt.2013.27

10. Ziesenitz VC, König SK, Mahlke NS, Skopp G, Haefeli WE, Mikus G (2015) Pharmacokinetic interaction of intravenous fentanyl with ketoconazole. The Journal of Clinical Pharmacology 55 (6):708-717

11. Eichbaum C, Cortese M, Blank A, Burhenne J, Mikus G (2013) Concentration effect relationship of CYP3A inhibition by ritonavir in humans. European journal of clinical pharmacology 69 (10):1795-1800

12. Stoll F, Burhenne J, Lausecker B, Weiss J, Thomsen T, Haefeli WE, Mikus G (2013) Reduced exposure variability of the CYP3A substrate simvastatin by dose individualization to CYP3A activity. The Journal of Clinical Pharmacology 53 (11):1199-1204
