# Supplementary material for: Composite midazolam and 1′-OH midazolam population pharmacokinetic model for constitutive, inhibited and induced CYP3A activity
Source: J Pharmacokinet Pharmacodyn. 2020 Aug 8;47(6):527–42. doi: 10.1007/s10928-020-09704-1 (PMC7652802; doi:10.1007/s10928-020-09704-1)
Supplement: Supplementary file 2 — Supplementary file2 (PDF 340 kb) Fig. S1 Comparison of 2- and 3-Compartment Model Error Distributions [file 10928_2020_9704_MOESM2_ESM.pdf]

ORIGINAL PAPER

Composite midazolam and 1'-OH midazolam population pharmacokinetic model for constitutive, inhibited and induced CYP3A activity

Sabrina T. Wiebe[1,2], Andreas D. Meid[1], Gerd Mikus[1]

[1]Department of Clinical Pharmacology and Pharmacoepidemiology, University of Heidelberg, Im Neuenheimer Feld 410, 69120 Heidelberg, Germany

[2]Boehringer Ingelheim Pharma GmbH & Co. KG, Birkendorfer Str. 65, 88397 Biberach an der Riss, Germany

Correspondence: Professor Gerd Mikus MD, Department of Clinical Pharmacology and Pharmacoepidemiology, University of Heidelberg, Im Neuenheimer Feld 410, 69120 Heidelberg, Germany; Tel.: +4962 2156 8740; Fax: +4962 2156 4642; E-mail: gerd.mikus@med.uni-heidelberg.de

Adopted Composite Model Control Stream with Interaction

```
$SIZES          LVR=50 ; increase number of allowed etas+epsilons to 50
$PROBLEM        Combined MDZ+1-OH MDZ PopPK Model
$INPUT          C ID XTIME=TIME TAFD=DROP AMT DOSE CMT ANA DV LDV=DROP NDV
                MDV SEX AGE WT EVID RATE TRT VIS STDY OCC LLOQ
                LLOQ_Unit=DROP TRT3 STYP
$DATA           Model_Development_Set.csv IGNORE=C
$SUBROUTINE     ADVAN6 TOL=4
$MODEL          COMP(ABSORB) ; absorption compartment
                COMP(CENTRAL) ; central midazolam compartment
                COMP(PERIPH1) ; midazolam peripheral compartment 1
                COMP(PERIPH2) ; midazolam peripheral compartment 2
                COMP(METABOL) ; central metabolite compartment
                COMP(MET_PER) ; metabolite peripheral compartment 1
                COMP(MET_PER2) ; metabolite peripheral compartment 2

;;========================= PARAMETER DEFINITIONS =========================
;;--------------------------------- INTEROCCASION VARIABILITY ---------------------------------
; interoccastion variability for midazolam clearance via systemic metabolism
$ABBREVIATED REPLACE ETA(OCC_QMET)=ETA(,10 to 14)

; interoccastion variability for 1'-OH midazolam clearance
$ABBREVIATED REPLACE ETA(OCC_CLM)=ETA(,15 to 19)

; interoccastion variability for midazolam bioavailability
$ABBREVIATED REPLACE ETA(OCC_F1)=ETA(,20 to 24)
;;-----------------------------------------------------------------------------------
$PK
;;------------------------------- INTERACTION -------------------------------------------
INH1 = 0 ; reversible inhibition off
INH2 = 0 ; irreversible inhibition off
IND = 0   ; induction off

IF(TRT3.EQ.2) INH1 = 1 ; reversible inhibition effect turned on
IF(TRT3.EQ.5) INH2 = 1 ; irreversible inhibition effect turned on
IF(TRT3.EQ.3) IND = 1 ; induction effect turned on

;;--------------------------------- PK MODEL ---------------------------------
; - MDZ (ANA = 0) -
   TVVC          = THETA(1)+INH2*THETA(25)
   VC            = TVVC*EXP(ETA(2))      ; central midazolam compartment volume

   TVVP1         = THETA(2)
   VP1           = TVVP1*EXP(ETA(5))    ; midazolam peripheral compartment1 volume

   TVVP2         = THETA(3)
   VP2           = TVVP2                ; midazolam peripheral compartment2 volume

   TVQP1         = THETA(4)
   QP1           = TVQP1*EXP(ETA(6))    ; intercompartmental clearance for peripheral compartment 1
```

```
  TVQP2        = THETA(5)
  QP2          = TVQP2                ; intercompartmental clearance for peripheral compartment 2

  TVQMET       = THETA(10)+INH1*THETA(26)+INH2*THETA(27)+IND*THETA(28)
  QMET         = TVQMET*EXP(ETA(3)+ETA(OCC_QMET)) ; clearance of midazolam via metabolism,
                                      ; with DDI effects (inhibition as one effect), IIV, and IOV

  TVF1         = THETA(7)+INH1*THETA(29)+INH2*THETA(30)+IND*THETA(31)
  F1           = TVF1*EXP(ETA(7)+ETA(OCC_F1)) ; bioavailability of midazolam with DDI effects
                                      ; (inhibition as one effect), IIV and IOV

  FM = 1              ; systemic fraction metabolized, fixed to 1 due to almost complete metabolism
                      ; of midazolam

; - 1'-OH MDZ (ANA =1) -

 TVKMET        = THETA(8)+INH2*THETA(32)+IND*THETA(33)
 KMET          = TVKMET*EXP(ETA(8)) ; pre-systemic metabolism rate with DDI effects
                                    ; (inhibition as one effect) and IIV

 TVVMET        = THETA(9)
 VMET          = TVVMET*EXP(ETA(1)) ; metabolic central compartment volume

 TVCLM         = THETA(11)+INH1*THETA(34)+INH2*THETA(35)
 CLM           = TVCLM*EXP(ETA(4)+ETA(OCC_CLM)) ; metabolic clearance with DDI, IIV, and IOV

 TVVMP         = THETA(12)
 VMP           = TVVMP    ; volume of metabolic peripheral compartment 1

 TVQMP         = THETA(13)*(WT/70)**THETA(24)
 QMP           = TVQMP*EXP(ETA(9)) ; metabolic inter-compartmental clearance with IIV (with
                                   ; weight covariate, normalized to approximate mean weight)

 TVVMP2        = THETA(14)
 VMP2          = TVVMP2 ; volume of metabolic peripheral compartment 2

 TVQMP2        = THETA(15)
 QMP2          = TVQMP2 ; metabolic inter-compartmental clearance 2

 IF(ANA.EQ.0) KA = THETA(6)       ; absorption rate constant (ANA = 0 is midazolam)
 IF(ANA.EQ.1) KA = KMET           ; pre-systemic metabolism rate constant (ANA = 1 is metabolite)

 K23   = QP1/VC
 K32   = QP1/VP1
 K24   = QP2/VC
 K42   = QP2/VP2
 K56   = QMP/VMET
 K65   = QMP/VMP
 K57   = QMP2/VMET
 K75   = QMP2/VMP2
 K2M   = FM*QMET/VC
 KM0   = CLM/VMET
```

```
  S2=VC
  S5=VMET
;;======================== DIFFERENTIAL EQUATIONS ============================
$DES
  DADT (1) =  - KA*A(1) ; KA becomes KMET for metabolite
  DADT (2) =    KA*A(1) - K23*A(2) + K32*A(3) -  K24*A(2) + K42*A(4)    -   K2M*A(2)
  DADT (3) =                   K23*A(2) -  K32*A(3)
  DADT (4) =                                                 K24*A(2) - K42*A(4)
  DADT (5) =    KMET*A(1) - K57*A(5) + K75*A(7) - K56*A(5) + K65*A(6) + K2M*A(2) - KM0*A(5)
  DADT (6) =                                                    K56*A(5) -  K65*A(6)
  DADT (7) =                        K57*A(5) -  K75*A(7)
;;============================== MODEL FIT ==================================
$ERROR

IPRED = F

IPRED = A(2)/S2                         ; IPRED for Analyte (ANA) = 0 (Midazolam)
IF(ANA.EQ.1) IPRED = A(5)/S5    ; IPRED for Analyte (ANA) = 1 (1'-OH metabolite)

FPROP1 = THETA(16) ; proportional residual error (midazolam early, no inhibition)
FADD1   = THETA(17) ; additive residual error (midazolam early, no inhibition)
FPROP2 = THETA(18) ; proportional residual error (1'-OH midazolam early, no inhibition)
FADD2   = THETA(19) ; additive residual error (1'-OH midazolam early, no inhibition)
FPROP3 = THETA(20) ; proportional residual error (midazolam late, no inhibition)
FADD3   = THETA(21) ; additive residual error (midazolam late, no inhibition)
FPROP4 = THETA(22) ; proportional residual error (1'-OH midazolam late, no inhibition)
FADD4   = THETA(23) ; additive residual error (1'-OH midazolam late, no inhibition)
FPROP5 = THETA(36) ; proportional residual error (both analytes early, with inhibition)
FADD5   = THETA(37) ; additive residual error (both analytes early, with inhibition)
FPROP6 = THETA(38) ; proportional residual error (both analytes late, with inhibition)
FADD6   = THETA(39) ; additive residual error (both analytes late, with inhibition)

; analytes not separated for early and late times following inhibition, as RUV error estimates were very
; similar

; Weighting factor for error for midazolam at early time points (all conditions but inhibition)
IF(TRT.NE.2.AND.ANA.EQ.0.AND.TIME.LE.0.5)  W = SQRT((FPROP1**2)*(IPRED**2)+(FADD1**2))

; Weighting factor for error for midazolam at later time points (all conditions but inhibition)
IF(TRT.NE.2.AND.ANA.EQ.0.AND.TIME.GT.0.5) W = SQRT((FPROP3**2)*(IPRED**2)+(FADD3**2))

; Weighting factor for error for 1'-OH midazolam at early time points (all conditions but inhibition)
IF(TRT.NE.2.AND.ANA.EQ.1.AND.TIME.LE.0.5)  W = SQRT((FPROP2**2)*(IPRED**2)+(FADD2**2))

; Weighting factor for error for 1'-OH midazolam at later time points (all conditions but inhibition)
IF(TRT.NE.2.AND.ANA.EQ.1.AND.TIME.GT.0.5) W = SQRT((FPROP4**2)*(IPRED**2)+(FADD4**2))

; Weighting factor for error for inhibition treatment at early time points (both analytes)
IF(TRT.EQ.2.AND.TIME.LE.1.5) W = SQRT((FPROP5**2)*(IPRED**2)+(FADD5**2))
```

```
; Weighting factor for error for inhibition treatment at later time points (both analytes)
IF(TRT.EQ.2.AND.TIME.GT.1.5) W = SQRT((FPROP6**2)*(IPRED**2)+(FADD6**2))

Y1  =  IPRED+W*EPS(1)       ; MDZ prediction [Proportional error]
Y2  =  IPRED+W*EPS(2)        ; 1'-OH MDZ prediction [Proportional error + small fixed additive error]

IRES= DV - IPRED
IWRES = IRES/W

Y = ANA*Y2+(1-ANA)*Y1 ; Combine both analytes for predictions
IF(ICALL.EQ.4.AND.Y.LE.0) Y=0.0001 ; for facilitating log-scale graphical display of VPC
;;========================== INITIAL ESTIMATES ==============================
$THETA
19.4507 FIX      ; VC
 41.0258 FIX     ; VP1
 23.8228 FIX     ; VP2
 8.00001 FIX     ; QP1
 46.0527 FIX     ; QP2
 2.30635 FIX     ; KA
 0.275824 FIX    ; F
 5.31099 FIX     ; KMET
 175.643 FIX     ; VMET
 24.1486 FIX     ; QMET
 196.827 FIX     ; CLM
 684.933 FIX     ; VMP
 59.5749 FIX     ; QMP
 67.145 FIX      ; VMP2
 127.374 FIX     ; QMP2
 0.503162 FIX    ; FPROP1
 0 FIX           ; FADD1
 0.555696 FIX    ; FPROP2
 0.00001 FIX     ; FADD2
 0.148938 FIX    ; FPROP3
 0 FIX           ; FADD3
 -0.215375 FIX   ; FPROP4
 0.00001 FIX     ; FADD4
 0.9863 FIX      ; QMP~WT
 51.8404         ; VC~INH2
 -16.336         ; QMET~INH1
-12.67           ; QMET~INH2
 37.9834         ; QMET~IND
 0.378806        ; F~INH1
 1.34248         ; F~INH2
 -0.200032       ; F~IND
 -5.23662        ; KMET~INH2
 12.4264         ; KMET~IND
 -64.1022        ; CLM~INH1
 1117.36         ; CLM~INH2
0.482949         ; FPROP5
 0 FIX           ; FADD5
 -0.267324       ; FPROP6
 0 FIX           ; FADD6
```

```
$OMEGA  BLOCK(2) FIX
 0.158449  ;   IIV_VMET
 0.109819 0.142583  ;   IIV_VC

$OMEGA
 0.0087203  FIX  ; IIV_QMET
 0.0176367  FIX  ; IIV_CLM
 0.167708  FIX    ; IIV_VP1
 0.251747  FIX    ; IIV_QP1
 0.0545658  FIX  ; IIV_F
 0.135776  FIX    ; IIV_KMET
 0.235254  FIX    ; IIV_QMP

$OMEGA  BLOCK(1) FIX
 0.0231946  ; IOV_QMET
$OMEGA  BLOCK(1) SAME
$OMEGA  BLOCK(1) SAME
$OMEGA  BLOCK(1) SAME
$OMEGA  BLOCK(1) SAME

$OMEGA  BLOCK(1) FIX
 0.0859661  ; IOV_CLM
$OMEGA  BLOCK(1) SAME
$OMEGA  BLOCK(1) SAME
$OMEGA  BLOCK(1) SAME
$OMEGA  BLOCK(1) SAME

$OMEGA  BLOCK(1) FIX
 0.0261532  ; IOV_F
$OMEGA  BLOCK(1) SAME
$OMEGA  BLOCK(1) SAME
$OMEGA  BLOCK(1) SAME
$OMEGA  BLOCK(1) SAME

$SIGMA
 1  FIX  ;   MDZ RUV
 1  FIX  ;   1-OH MDZ RUV
;;======================== ESTIMATION METHOD ===============================
$ESTIMATION   METHOD=1   INTERACTION   MAXEVAL=9999   NOABORT   SADDLE_RESET=2   PRINT=5
NSIG=3 ; FOCE-I
$COVARIANCE PRINT=E PRECOND=2

;;=============================== TABLES ====================================
$TABLE          ANA ID TIME AMT CMT MDV EVID RATE SEX AGE WT STDY VIS
                TRT STYP ETAS(1:LAST) IPRED IWRES CIPRED CPRED
                CRES CWRES CIWRES NOPRINT ONEHEADER FILE=sdtab1

$TABLE          ANA ID STDY CLM VC VP1 VP2 VMET VMP VMP2 QP1 QP2 QMET QMP QMP2 KA
                F1 KMET TRT SEX AGE WT FPROP1 FPROP2 FPROP3 FPROP4 FPROP5 FPROP6
                ETAS(1:LAST) IPRED IWRES CIPRED CPRED CRES
                CWRES CIWRES NOPRINT ONEHEADER FILE=patab1
```
