# Supplementary material for: Composite midazolam and 1′-OH midazolam population pharmacokinetic model for constitutive, inhibited and induced CYP3A activity
Source: J Pharmacokinet Pharmacodyn. 2020 Aug 8;47(6):527–42. doi: 10.1007/s10928-020-09704-1 (PMC7652802; doi:10.1007/s10928-020-09704-1)
Supplement: Supplementary file 3 — Supplementary file3 (PDF 594 kb) Fig. S2 Composite Midazolam Interaction Model VPC – External Validation [file 10928_2020_9704_MOESM3_ESM.pdf]

ORIGINAL PAPER

Composite midazolam and 1'-OH midazolam population pharmacokinetic model for constitutive, inhibited and induced CYP3A activity

Sabrina T. Wiebe[1,2], Andreas D. Meid[1], Gerd Mikus[1]

[1]Department of Clinical Pharmacology and Pharmacoepidemiology, University of Heidelberg, Im Neuenheimer Feld 410, 69120 Heidelberg, Germany

[2]Boehringer Ingelheim Pharma GmbH & Co. KG, Birkendorfer Str. 65, 88397 Biberach an der Riss, Germany

Correspondence: Professor Gerd Mikus MD, Department of Clinical Pharmacology and Pharmacoepidemiology, University of Heidelberg, Im Neuenheimer Feld 410, 69120 Heidelberg, Germany; Tel.: +4962 2156 8740; Fax: +4962 2156 4642; E-mail: gerd.mikus@med.uni-heidelberg.de

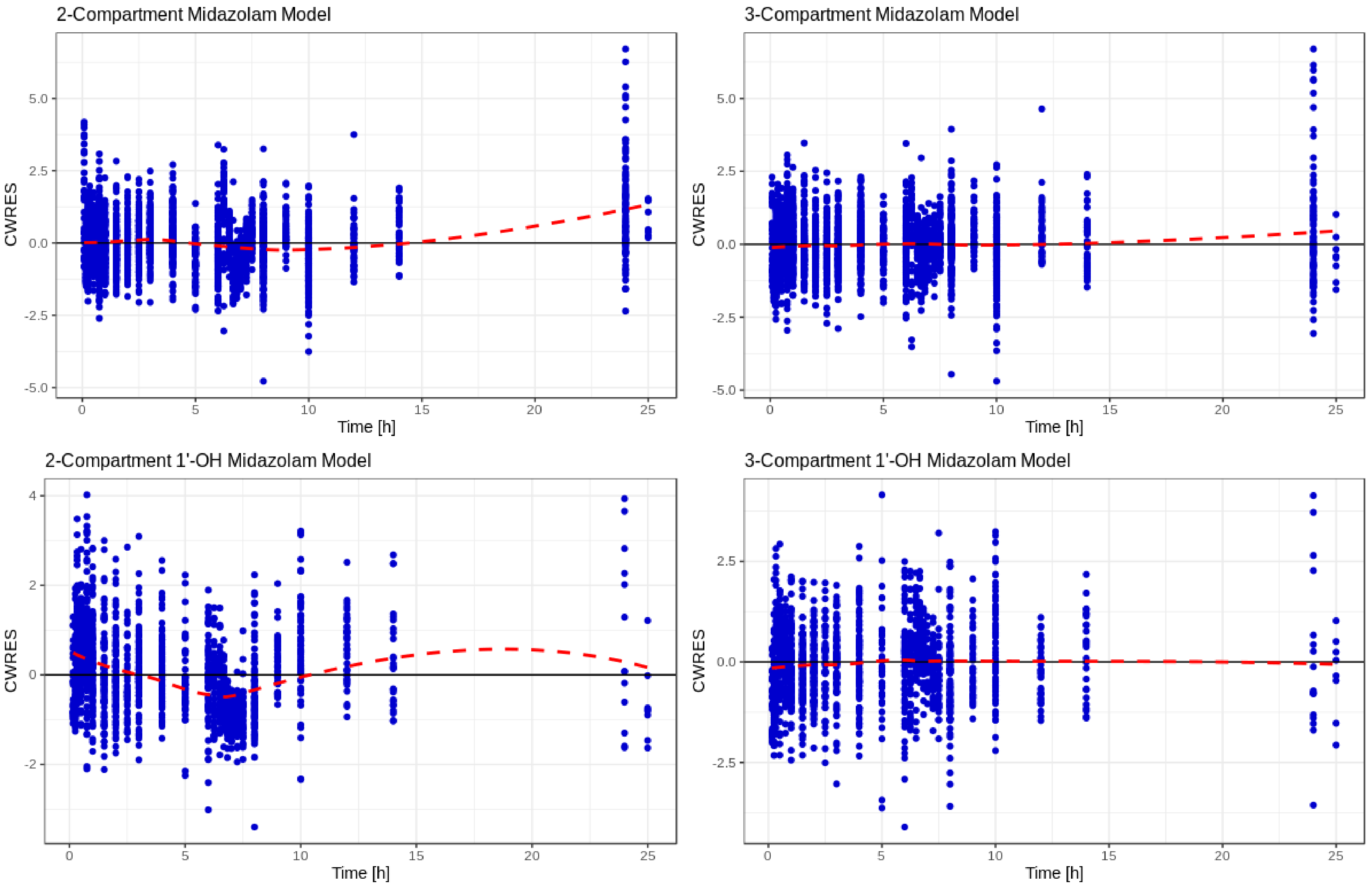

Fig. S1 Comparison of the distribution of conditional weighted errors over time for the 2-compartment and 3-compartment models for midazolam (top) and 1’-OH midazolam (bottom). The dashed line is based on a loess smoothing function. CWRES = conditional weighted residual error
