# Supplementary material for: Composite midazolam and 1′-OH midazolam population pharmacokinetic model for constitutive, inhibited and induced CYP3A activity
Source: J Pharmacokinet Pharmacodyn. 2020 Aug 8;47(6):527–42. doi: 10.1007/s10928-020-09704-1 (PMC7652802; doi:10.1007/s10928-020-09704-1)
Supplement: Supplementary file 4 — Supplementary file4 (PDF 661 kb) Figs. S3-S6 Sampling Importance Resampling of Composite and Interaction Models [file 10928_2020_9704_MOESM4_ESM.pdf]

ORIGINAL PAPER

Composite midazolam and 1'-OH midazolam population pharmacokinetic model for constitutive, inhibited and induced CYP3A activity

Sabrina T. Wiebe[1,2], Andreas D. Meid[1], Gerd Mikus[1]

[1]Department of Clinical Pharmacology and Pharmacoepidemiology, University of Heidelberg, Im Neuenheimer Feld 410, 69120 Heidelberg, Germany

[2]Boehringer Ingelheim Pharma GmbH & Co. KG, Birkendorfer Str. 65, 88397 Biberach an der Riss, Germany

Correspondence: Professor Gerd Mikus MD, Department of Clinical Pharmacology and Pharmacoepidemiology, University of Heidelberg, Im Neuenheimer Feld 410, 69120 Heidelberg, Germany; Tel.: +4962 2156 8740; Fax: +4962 2156 4642; E-mail: gerd.mikus@med.uni-heidelberg.de

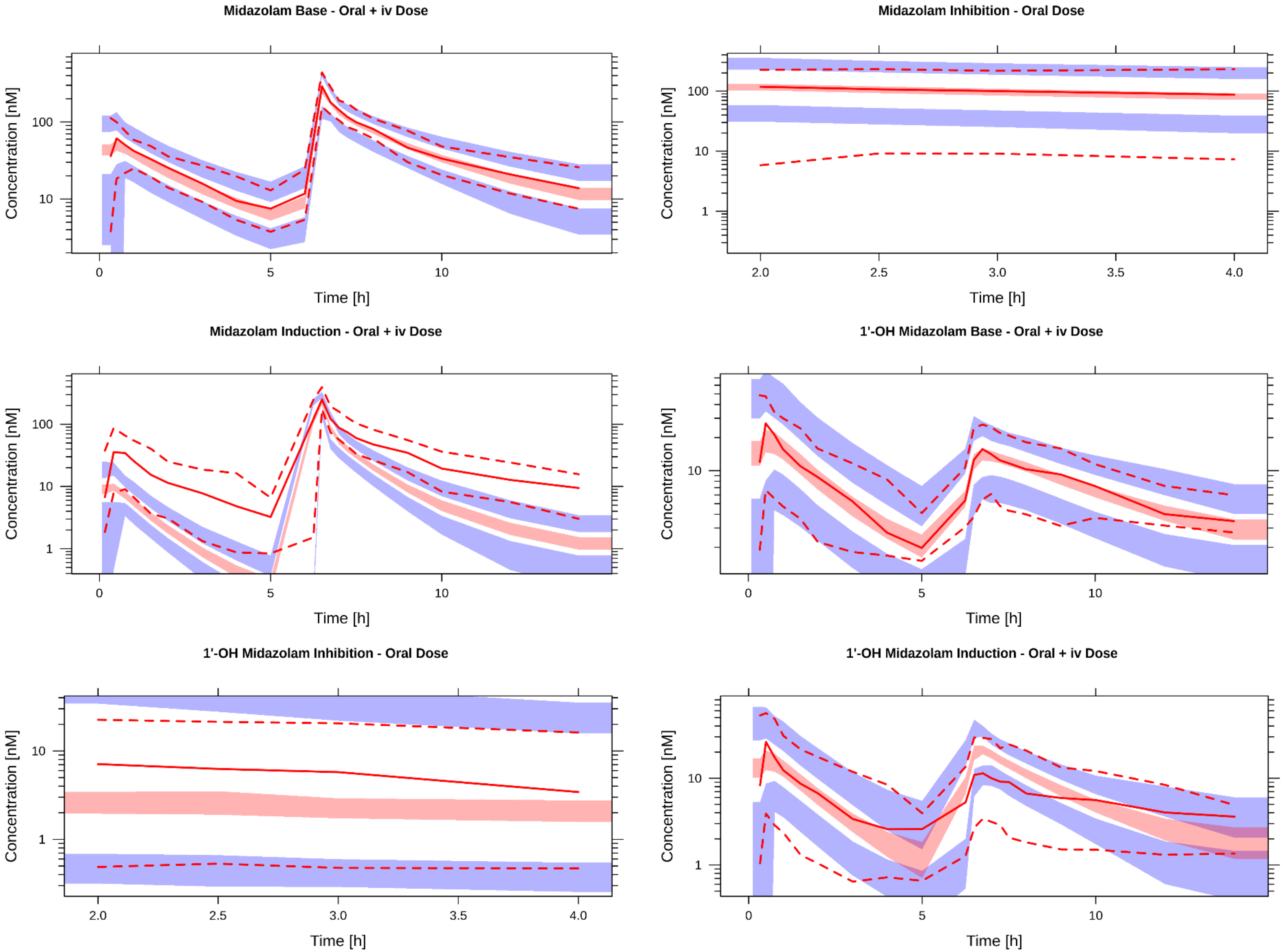

Fig. S2 Visual predictive checks (1000 simulations) for the external validation set using the final interaction model. Solid red lines depict the observed median concentrations; red dotted lines depict the observed 97.5th and 2.5th percentiles. The red area pertains to the 90% confidence interval for the predicted medians, while the blue areas pertain to the 90% confidence interval for the 97.5th and 2.5th percentile predictions. Data are normalized to a midazolam dose of 4 mg (semi-log scale)
