# Supplementary material for: Composite midazolam and 1′-OH midazolam population pharmacokinetic model for constitutive, inhibited and induced CYP3A activity
Source: J Pharmacokinet Pharmacodyn. 2020 Aug 8;47(6):527–42. doi: 10.1007/s10928-020-09704-1 (PMC7652802; doi:10.1007/s10928-020-09704-1)
Supplement: Supplementary file 5 — Supplementary file5 (PDF 1369 kb) [file 10928_2020_9704_MOESM5_ESM.pdf]

ORIGINAL PAPER

Composite midazolam and 1'-OH midazolam population pharmacokinetic model for constitutive, inhibited and induced CYP3A activity

Sabrina T. Wiebe[1,2], Andreas D. Meid[1], Gerd Mikus[1]

[1]Department of Clinical Pharmacology and Pharmacoepidemiology, University of Heidelberg, Im Neuenheimer Feld 410, 69120 Heidelberg, Germany

[2]Boehringer Ingelheim Pharma GmbH & Co. KG, Birkendorfer Str. 65, 88397 Biberach an der Riss, Germany

Correspondence: Professor Gerd Mikus MD, Department of Clinical Pharmacology and Pharmacoepidemiology, University of Heidelberg, Im Neuenheimer Feld 410, 69120 Heidelberg, Germany; Tel.: +4962 2156 8740; Fax: +4962 2156 4642; E-mail: gerd.mikus@med.uni-heidelberg.de

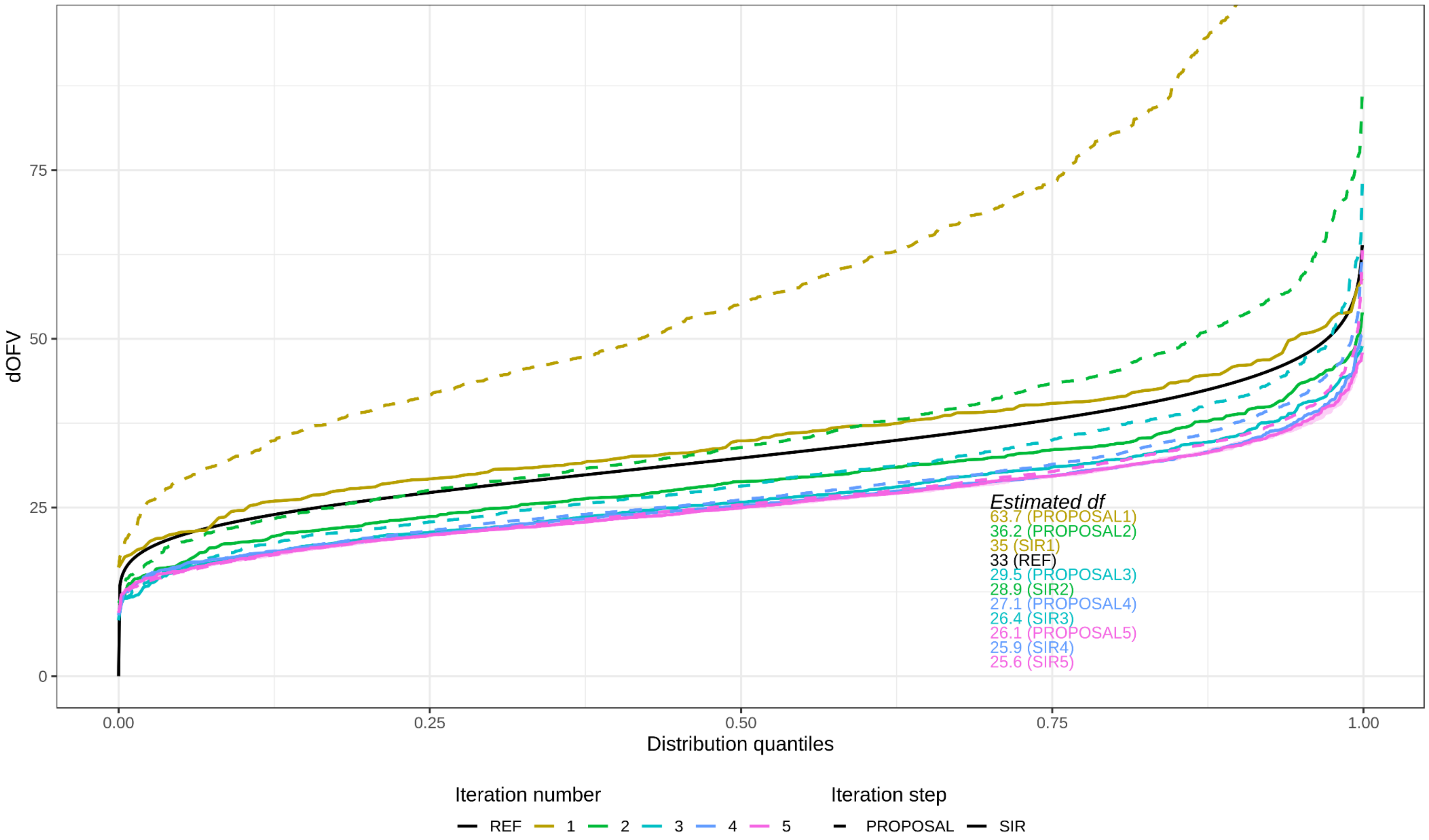

Fig. S3 Delta Objective Function Values (dOFV) for all Composite Model iterations following Sampling Importance Re-sampling (SIR). The black line is the reference Chi Square distribution, dashed lines are the proposal densities used for importance weighting, and solid lines represent the outcomes of the SIR

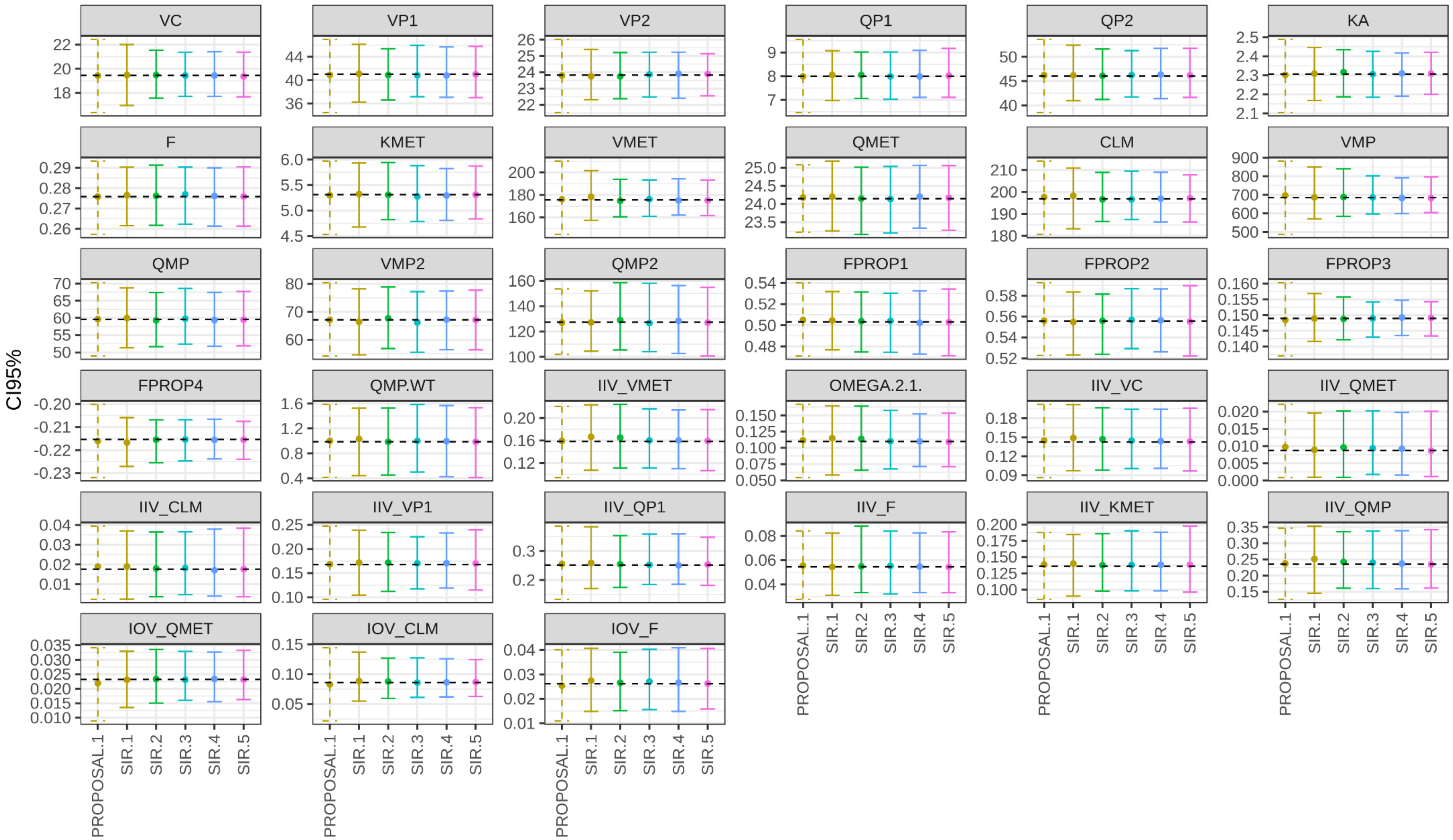

Fig. S4 Confidence intervals for each of the Composite Model parameters over all iterations based on SIR analyses. The proposal represents intervals based on the covariance-matrix from NONMEM

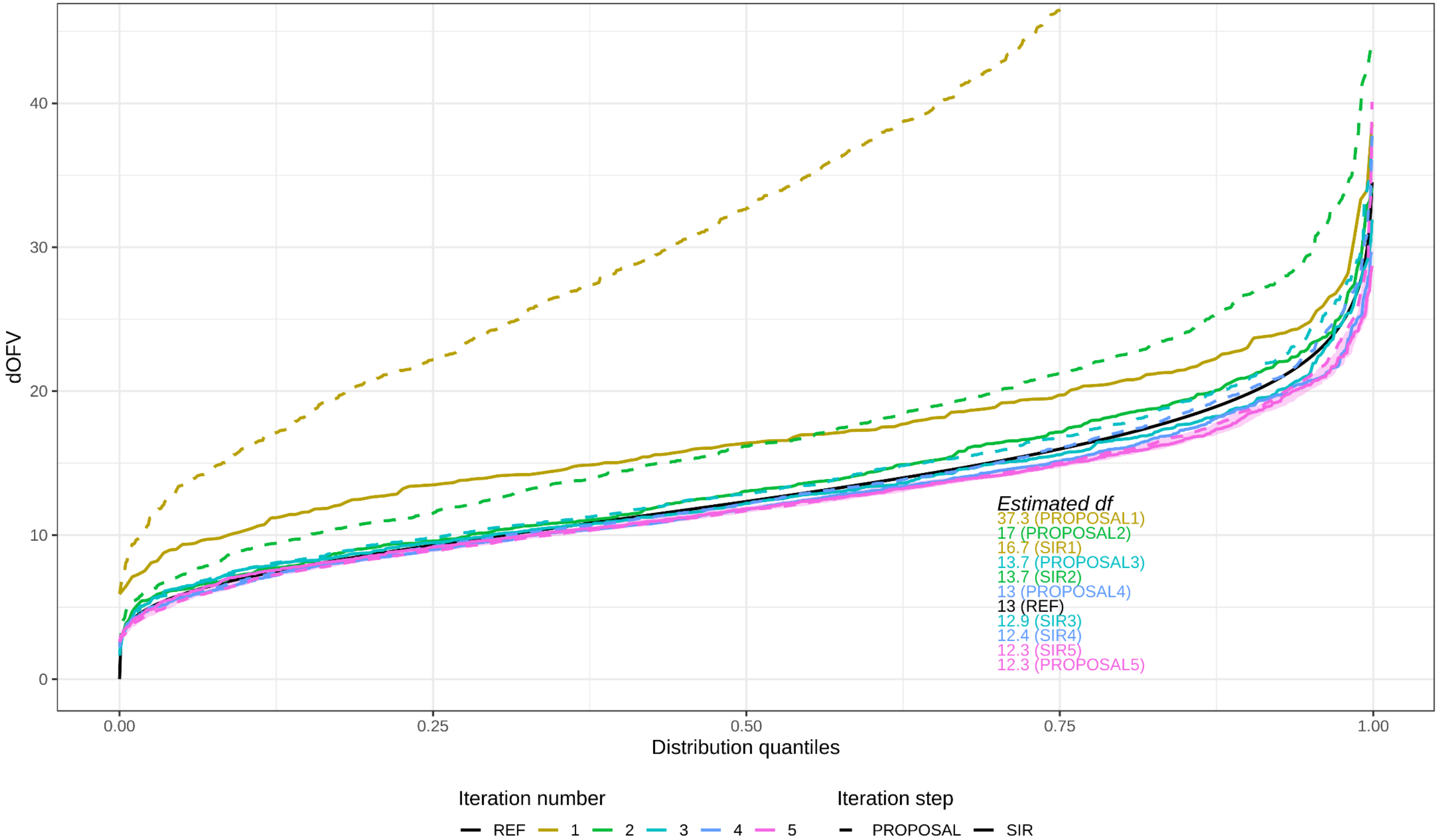

Fig. S5 Delta Objective Function Values (dOFV) for all Interaction Model iterations following Sampling Importance Re-sampling (SIR). The black line is the reference Chi Square distribution, dashed lines are the proposal densities used for importance weighting, and solid lines represent the outcomes of the SIR

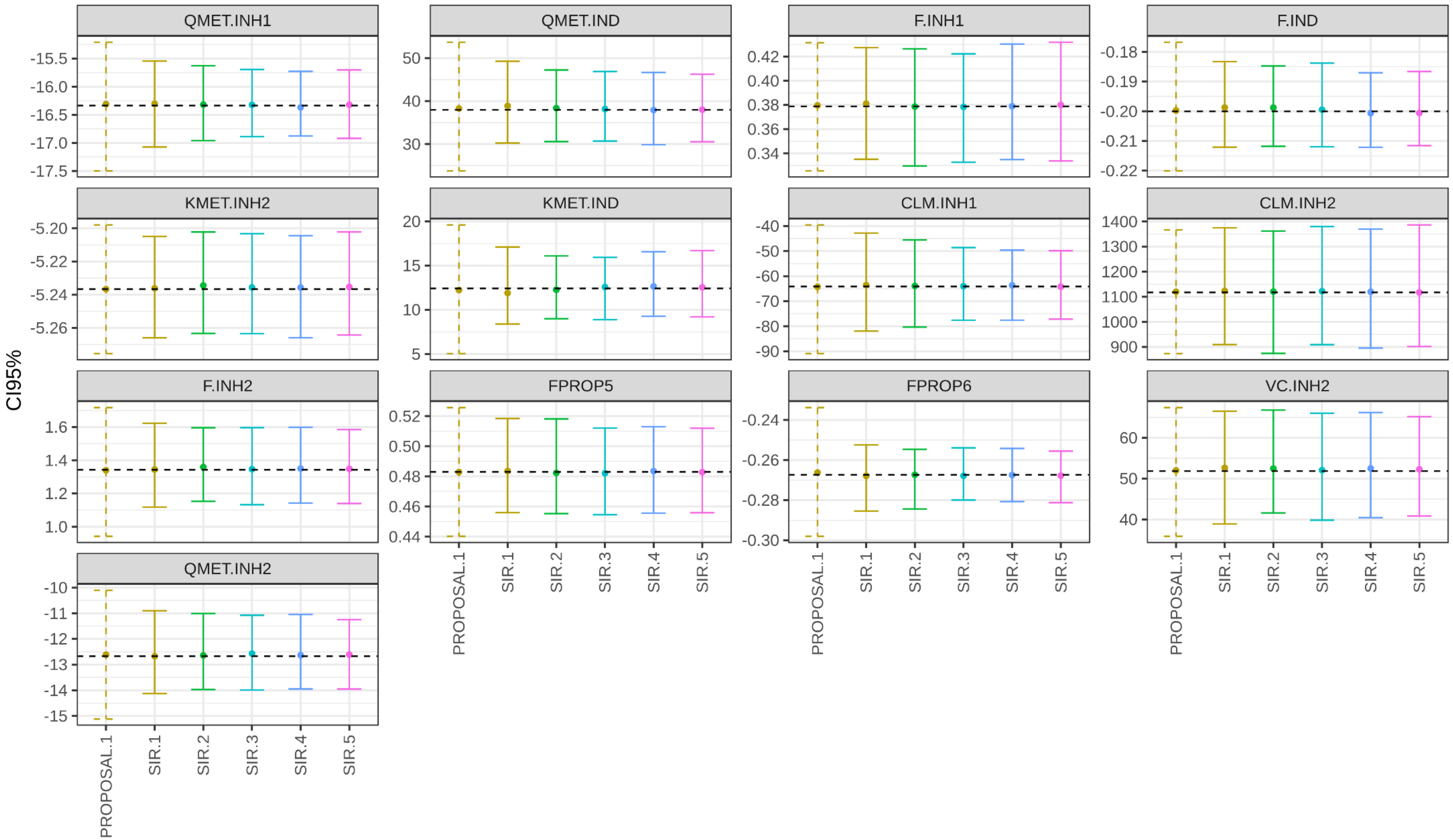

Fig. S6 Confidence intervals for each of the Interaction Model parameters over all iterations based on SIR analyses. The proposal represents intervals based on the covariance-matrix from NONMEM
